# Visual fixation patterns during economic choice reflect covert valuation processes that emerge with learning

**DOI:** 10.1073/pnas.1906662116

**Published:** 2019-10-21

**Authors:** Sean E. Cavanagh, W. M. Nishantha Malalasekera, Bruno Miranda, Laurence T. Hunt, Steven W. Kennerley

**Affiliations:** ^a^Department of Clinical and Movement Neurosciences, University College London, WC1N 3BG London, United Kingdom;; ^b^International Neuroscience Doctoral Programme, Champalimaud Foundation, 1400-038 Lisbon, Portugal;; ^c^Instituto de Medicina Molecular, Faculdade de Medicina, Universidade de Lisboa, 1649-028 Lisbon, Portugal;; ^d^Wellcome Trust Centre for Neuroimaging, University College London, WC1N 3BG London, United Kingdom;; ^e^Max Planck University College London Centre for Computational Psychiatry and Ageing Research, University College London, WC1B 5EH London, United Kingdom;; ^f^Wellcome Centre for Integrative Neuroimaging, Department of Psychiatry, University of Oxford, OX3 7JX Oxford, United Kingdom

**Keywords:** decision making, value, attention, learning, novelty bonus

## Abstract

Visual fixations play a vital role in decision making. Recent studies have demonstrated that the longer subjects fixate an option, the more likely they are to choose it. However, the role of evaluating stimuli covertly (i.e., without fixating them), and how covert evaluations determine where to subsequently fixate, remains relatively unexplored. Here, we trained monkeys to perform a decision-making task where they made binary choices between reward-predictive stimuli which were well-learned (“overtrained”), recently learned (“novel”), or a combination of both (“mixed”). Subjects were free to saccade around the screen and make a choice (via joystick response) at any time. Subjects rarely fixated both options, yet choice behavior was better explained by assuming the values of both stimuli governed choices. The first fixation latency was fast (∼150 ms) but, surprisingly, its direction was value-driven. This suggests covert evaluation of stimulus values prior to first saccade. This was particularly evident for overtrained stimuli. For novel stimuli, first fixations became increasingly value-driven throughout a behavioral session. However, this improvement lagged behind learning of accurate economic choices, suggesting separate processes governed their learning. Finally, mixed trials revealed a strong bias toward fixating the novel stimulus first but no bias toward choosing it. Our results suggest that the primate brain contains fast covert evaluation mechanisms for guiding fixations toward highly valuable and novel information. By employing such covert mechanisms, fixation behavior becomes dissociable from the value comparison processes that drive final choice. This implies that primates use separable decision systems for value-guided fixations and value-guided choice.

## Significance

Where we direct our gaze can have a big impact on what we choose. However, where we choose to gaze during the decision process is not well-characterized, despite the important role it plays. In our study, monkeys performed a simple decision-making experiment where they were free to look around a computer screen showing choice options. They then indicated their economic choice with a joystick movement. When choice options appeared, monkeys rapidly gazed toward more valuable and novel stimuli—suggesting there is a system that orients gaze toward important information. However, despite the gaze preference for novel stimuli, subjects did not prefer to choose them. This suggests the mechanisms governing value-guided attentional capture and value-guided choice are dissociable.

We constantly experience a rich assortment of visual information, some of which is highly relevant to future decisions. To make decisions efficiently, we must quickly identify relevant information in our environment—a process often accomplished by orienting our eyes toward this information (i.e., visual fixations).

Human economic decision-making experiments have highlighted the significance of fixation patterns in guiding subsequent choices. When deliberating between multiple items, participants tend to fixate longer upon items they will ultimately choose (1–4). Furthermore, an experimenter can bias a participant’s choices by manipulating what they fixate and for how long (5). Computational models designed to explain these findings have been highly influential, and can predict choice accuracy and reaction time distributions when provided with fixation data (2, 3). However, these models do not typically attempt to explain the processes which determine where participants decide to fixate. In the real world, we often experience visual scenes with far too many options available to fixate prior to making a choice (6). To make effective choices, it is key that we can identify stimuli we might want to choose before fixating them. We will refer to the mechanism by which visual stimuli located outside of the fovea are processed as “covert evaluation.” The degree to which covert evaluation influences how we sample information with fixations during choices is thus an important unresolved question, as is to what degree covert evaluation influences actual choice.

Outside of the economic choice literature, it has often been shown that basic stimulus features (e.g., color, intensity, orientation) have a powerful influence on attention and fixation behavior (7–11). For instance, salient distractors attract fixations (10, 11) and delay visual search (7). Recently, evidence for a similar distracting effect by nonphysical stimulus properties has been revealed; stimuli previously associated with reward also have a distracting influence during visual search (12–14), and attract fixations in free-viewing settings (15–17).

While there has been interest in the role of low-level stimulus features (e.g., color, size) in influencing fixation patterns during economic choices (18, 19), the role of stimulus value is not well-characterized. Some studies suggest first fixations during choice are not influenced by stimulus value, and are instead determined randomly (1–3). Therefore, these studies appear to find diametrically opposite results to what has been shown in the attentional-capture literature (12–17). It is currently unknown why stimulus value influences fixation deployment in one set of experiments (12–17) but has no influence in other experiments (1–3). An important difference between these 2 sets of studies is the subjects’ familiarity with the stimuli. One possibility is that fixations can be influenced by the value of familiar stimuli during economic choices, but that previous studies did not observe this because they only utilized relatively novel stimuli.

To investigate the effect of stimulus familiarity and value on fixation patterns during economic choice, we recorded eye-position data during a free-viewing decision-making task. Nonhuman primates made binary choices between differently valued stimuli that were either well-learned (experienced over 100 times prior to testing) or recently learned (learned that day prior to testing). On choices between well-learned stimuli, subjects used covert evaluation to guide their first fixation toward the option with greater value within ∼150 ms. On choices between recently learned stimuli, first fixations were initially relatively random, even though subjects made accurate economic choices early in learning. These findings suggest the primate brain contains fast covert valuation mechanisms to bias fixations toward valuable information, yet the systems supporting value-guided fixations and value-guided choice dissociate with stimulus familiarity.

## Results

**Fig. 1.**
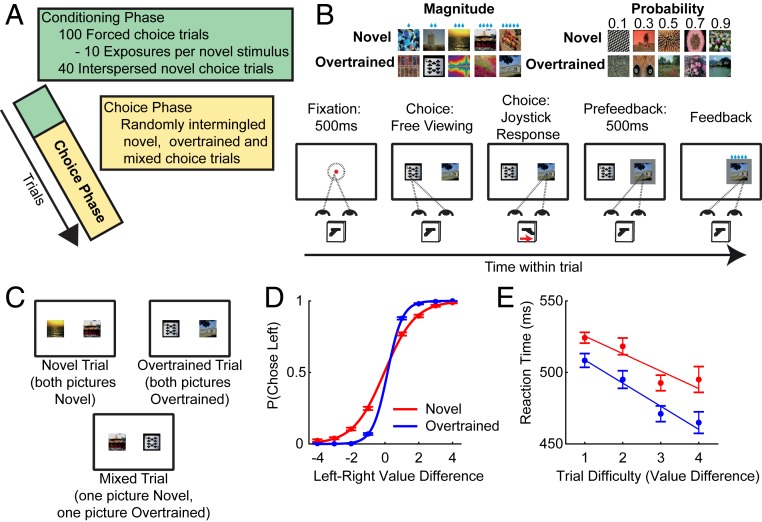
Task and performance. (*A*) Session structure. Sessions began with a conditioning phase where subjects completed 10 1-alternative forced-choice trials of 10 novel stimuli to learn their values (*SI Appendix*, Fig. S1). Subjects then entered the choice phase, where they made 2-alternative choices. (*B*) Choice phase. Subjects were shown 2 cues; they were free to saccade between them and make a joystick response to indicate their choice at any time. (*B*, *Top*) An example cue set. Cues could indicate reward magnitude or probability, and could either be well-known to subjects (overtrained) or novel (learned in the conditioning phase). (*C*) Example trials. On any given trial, subjects could be presented with only overtrained cues (overtrained trials), novel cues (novel trials), or 1 of each (mixed trials). Subjects were only presented choices within an attribute dimension (i.e., 2 probability or 2 magnitude cues). (*D*) Choice performance (±SE) as a function of the difference between stimulus values. (*E*) Reaction time (±SEM) as a function of trial difficulty. Subjects made decisions more quickly on easier trials.

Two macaque monkeys (F and M) were trained to perform a decision-making task (Fig. 1) in which they selected between 2 overtrained (well-learned) or novel (learned that day) stimuli with 5 discrete values (*SI Appendix*, *Text*). Crucially, following stimulus onset, subjects were free to saccade around the screen and make a choice using a joystick movement. Each day, prior to performing the free-choice task, they completed 100 conditioning trials (10 trials for each stimulus) in which they learned the reward probability and reward magnitude predicted by the novel stimuli (*SI Appendix*, *Text* and Fig. S1). Subject F performed 20 sessions completing 11,649 choice trials, while subject M performed 14 sessions completing 9,518 choice trials. Trials were pseudorandomly selected from 1 of 3 conditions: novel, overtrained, or mixed (Fig. 1*C*). Main-text figures show data collapsed across subjects (*SI Appendix*, Figs. S2–S14 show data for each subject).

### Choice Behavior.

Subjects were proficient at selecting the more valuable option (Fig. 1*D* and *SI Appendix*, Fig. S2 and Table S1). A logistic regression of choice side against left-minus-right value difference found a strong value-based effect (Fig. 1*D* and *SI Appendix*, Fig. S2). Choices were more sensitive to value in overtrained than in novel trials [linear hypothesis test of β_(Overtrained)_ > β_(Novel)_: *P* < 10^−10^ for both subjects; Fig. 1*D* and *SI Appendix*, Fig. S2]. Reaction time was also influenced by value, with faster responses recorded on easier trials (Fig. 1*E* and *SI Appendix*, Fig. S2). This suggests subjects made decisions by comparing values, as humans also do in economic choice tasks.

### Value Influences First Fixation Direction, within 150 ms of Stimulus Onset.

**Fig. 2.**
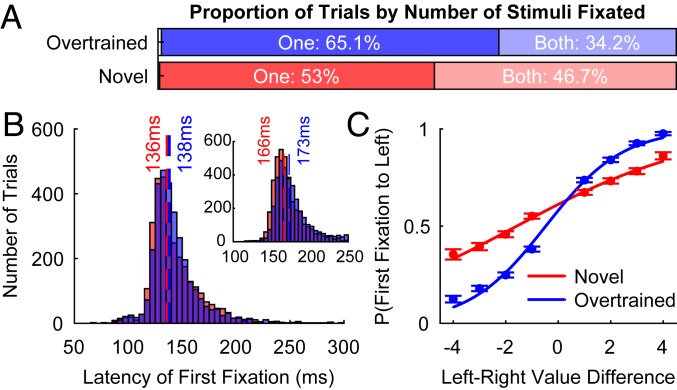
Subjects rapidly fixate more valuable stimuli. (*A*) The proportion of trials where subjects viewed 0, 1, or both stimuli. (*B*) Histograms of the latency with which subjects fixated the first stimulus in novel (red) and overtrained (blue) trials. Both subjects rapidly fixate a stimulus within ∼150 ms on most trials. The main plot displays data for subject M; the *Inset* shows data for subject F. Dashed vertical lines show the median latency per trial type. (*C*) The probability (±SE) of fixating the left stimulus first as a function of left–right stimulus value difference. First fixations were generally to the more valuable stimulus. This effect is stronger on overtrained than on novel trials. Lines show logistic model fits.

As subjects were free to view the stimuli, we examined whether fixations were influenced by the value of the stimuli. We analyzed the eye-position data using an algorithm to detect saccades that resulted in the fixation of a stimulus (*SI Appendix*, *Text*). This revealed that subjects almost always fixated at least 1 stimulus prior to choosing with the joystick (F and M: >99%; Fig. 2*A* and *SI Appendix*, Fig. S3) and their first fixations occurred shortly after stimulus onset (Fig. 2*B*; median latency: F: 169 ms; M: 137 ms). Surprisingly, the direction of this first fixation was not random but was strongly influenced by the value of the stimuli. For both novel and overtrained trials, a logistic regression of fixation direction against left-minus-right value difference revealed that stimulus value significantly influenced which stimulus was fixated first (Fig. 2*C* and *SI Appendix*, Fig. S3), even when the best stimulus was available (*SI Appendix*, Fig. S4). As with subjects’ eventual choices, this effect was stronger in overtrained than novel trials [linear hypothesis test, β_(Overtrained)_ > β_(Novel)_: *P* < 10^−10^ for both subjects; Fig. 2*C*]. This result implies that subjects were performing reliable covert stimulus evaluations within ∼150 ms of stimulus onset, which guided their first fixation toward the more valuable stimulus. It also suggested that subjects’ optimality in this evaluation varied dependent upon how familiar they were with the stimuli. However, the latency of fixation was not clearly influenced by stimulus value (*SI Appendix*, Fig. S3).

**Fig. 3.**
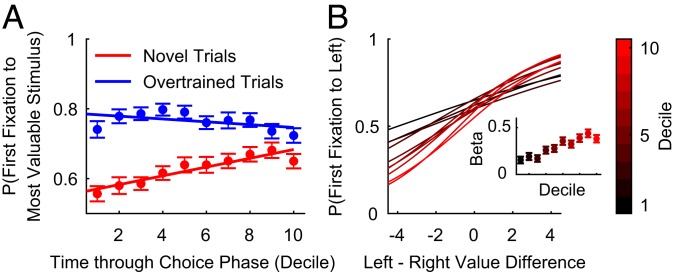
Subjects learn to direct their first fixation toward more valuable novel stimuli during a session. (*A*) The mean (±SEM) proportion of trials where the subjects’ first fixation was toward the more valuable stimulus, as a function of session decile. On the earliest novel trials, subjects’ first fixation direction is not influenced by value. However, as the stimulus values are learned, subjects develop a preference to fixate the more valuable stimulus first. On overtrained trials, subjects consistently fixate the more valuable stimulus first. (*B*) Logistic fit of the probability of fixating the left stimulus first as a function of left–right stimulus value difference for novel trials. The data are split by session decile. (*B*, *Inset*) The regression coefficient (±SE) quantifying the effect of value on fixation direction in each trial decile. On novel trials, first fixation direction is increasingly influenced by stimulus values as the session progresses.

To explore how the likelihood of value-driven fixations increased with learning and experience, we correlated the probability of the first fixation being toward the most valuable stimulus within each trial decile across a session (Fig. 3*A* and *SI Appendix*, Fig. S5). In novel trials, both subjects showed a positive correlation—they became increasingly more likely to direct their first fixation to the more valuable stimulus as the session progressed (Spearman’s correlation: F: *r* = 0.2511, *P* = 3.354 × 10^−4^; M: *r* = 0.2805, *P* = 7.896 × 10^−4^). No such trend existed on overtrained trials (F: *r* = −0.0989, *P* = 0.1637; M: *r* = −0.0995, *P* = 0.2419), and there was a significant difference between novel and overtrained trials (Fisher’s test: F: *P* = 4.143 × 10^−4^; M: *P* = 0.0013). We further verified these results using logistic regression with first fixation direction as the dependent variable (Fig. 3*B* and *SI Appendix*, *Text* and Fig. S5). As novel trial decile number increased, the regression coefficient for (left-minus-right) value difference increased. This confirmed that stimulus value increasingly influenced first fixation direction as subjects gained more experience with each stimulus (Spearman’s correlation: F: *r* = 0.9152, *P* = 4.667 × 10^−4^; M: *r* = 0.8667, *P* = 0.0027). These results indicate that first fixations can be strongly influenced by value using covert mechanisms that emerge with limited experience. Importantly, however, while the likelihood of fixating the most valuable stimulus on novel trials increased across a session, the likelihood of choosing the most valuable stimulus on novel trials was already near-ceiling early in the session (*SI Appendix*, Fig. S6). Therefore, choice accuracy exhibited only modest to insignificant improvement (*SI Appendix*, Fig. S6). This implies a dissociation in the learning time courses for the neural systems which bias fixations and govern choices.

### Likelihood of Subsequent Fixations, and First Fixation Dwell Time, Reflects the Value of the Nonfixated Stimulus.

**Fig. 4.**
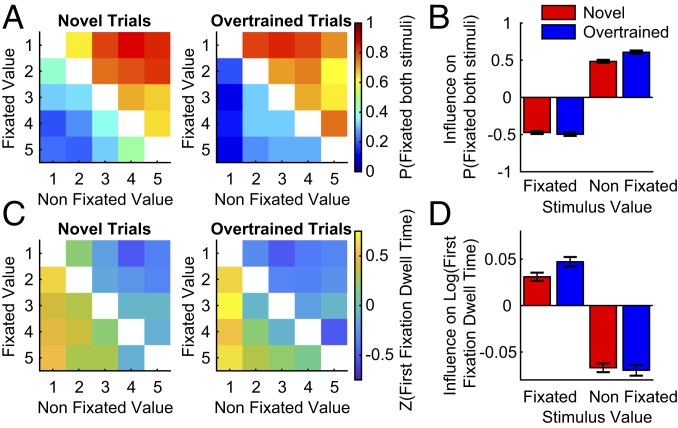
Subjects’ likelihood of fixating a second stimulus, and how long they dwell on their first fixation, is influenced by covert evaluation of the nonfixated stimulus. (*A*) The proportion of trials where the subjects fixated both stimuli, as a function of the first fixated and nonfixated stimulus values. (*B*) Logistic regression coefficients (±SE) of the probability of fixating both stimuli as a function of the first fixated and nonfixated stimulus values. (*C*) The *z*-scored dwell time on the first fixated stimulus on trials where both stimuli were viewed. The dwell time is a function of the fixated and nonfixated stimulus values. (*D*) Linear regression coefficients (±SE) of the log-transformed first stimulus dwell time as a function of fixated and nonfixated stimulus values.

First fixations tended to be toward more valuable stimuli, implying a covert valuation process driving fixation behavior. We next examined whether the propensity to fixate the second stimulus also reflected a covert valuation process. Unsurprisingly, the probability of fixating both stimuli was influenced negatively by the value of the first fixated stimulus. However, the probability of fixating both stimuli was also positively influenced by the value of the nonfixated stimulus (Fig. 4 *A* and *B* and *SI Appendix*, Fig. S7). In other words, the value of the second stimulus (which had not yet been fixated) had a strong influence on the likelihood of sampling more information. This was the case for both novel and overtrained trials, and irrespective of whether the first fixation was to the most valuable stimulus (*SI Appendix*, Fig. S7).

We next examined whether covert evaluation also influenced first fixation dwell time. On trials where both stimuli were fixated, there was a positive/negative influence of fixated/nonfixated stimulus value, respectively (Fig. 4 *C* and *D* and *SI Appendix*, Fig. S8). Thus, subjects tended to dwell on a stimulus for a shorter duration, irrespective of its value, as the value of the nonfixated stimulus increased. This provides further evidence that prior to fixating the second stimulus, subjects covertly processed its value. Coupled with the influence of value on first fixation direction, this demonstrates that information sampling is influenced by the values of yet-to-be fixated stimuli.

### Fixations, but Not Choices, Show a Novelty Bias.

**Fig. 5.**
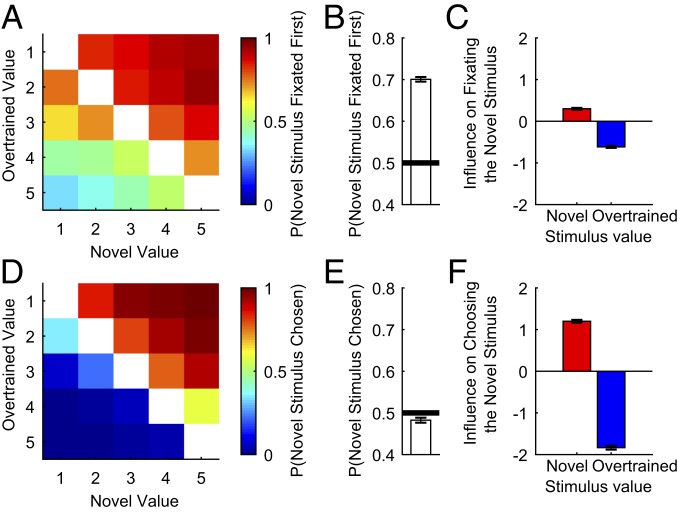
Dissociation between fixation and choice behavior on mixed trials. When choosing between a novel and overtrained stimulus, subjects prefer to fixate the novel stimulus first but not to choose it. (*A*) The proportion of trials where subjects viewed the novel stimulus first, as a function of novel and overtrained stimulus values. Subjects preferred to fixate the novel stimulus first, but fixations were still influenced by stimulus value. (*B*) Proportion of trials where subjects viewed the novel stimulus first. (*C*) Logistic regression coefficients for the influence of stimulus value on the probability of fixating the novel stimulus first. (*D*) The proportion of trials where subjects chose the novel stimulus, as a function of the novel and overtrained stimulus values. There is no preference for choosing the novel stimulus; choices are strongly influenced by value alone. (*E*) Proportion of trials where subjects chose the novel stimulus. (*F*) Logistic regression coefficients for the influence of stimulus value on the probability of choosing the novel stimulus. All error bars denote SE.

We next examined mixed trials, which contained 1 overtrained and 1 novel stimulus. As before, logistic regression showed a strong effect of both novel and overtrained stimulus value on first fixation direction (Fig. 5 *A* and *C* and *SI Appendix*, Fig. S9). However, an additional influence on first fixation direction was observed on mixed trials compared with overtrained-only or novel-only trials: First fixations were strongly biased toward the novel stimulus (Fig. 5 *A* and *B* and *SI Appendix*, Fig. S9; binomial test: F: *P* < 10^−10^; M: *P* < 10^−10^). However, unlike the value-driven effects on first fixation observed in novel trials (Fig. 3 and *SI Appendix*, Fig. S5), the novelty bias did not markedly change across the session (*SI Appendix*, Fig. S10).

This novelty bias shows subjects preferred to initially fixate novel stimuli, but did they also prefer to choose them? We tested this by repeating the same analyses on subjects’ choices (Fig. 5 *D*–*F* and *SI Appendix*, Fig. S9). There was no novelty bias in the subjects’ choices, and subject F had a slight preference for choosing the overtrained stimulus (Fig. 5 *D* and *E* and *SI Appendix*, Fig. S9; binomial test: F: *P* = 0.0146; M: *P* = 0.0974). This dissociation in the effect of novel stimuli on fixations versus economic choice provides further evidence of separate valuation and decision processes supporting each.

### The Role of Fixations and Covert Evaluations in Choice.

We have shown that the covert valuation processes that bias fixations are dissociable from the valuation processes that guide choices. However, recent decision-making models indicate that—over and above the value-related effects we observed—how long a stimulus is fixated should influence its likelihood of being chosen (2, 3).

**Fig. 6.**
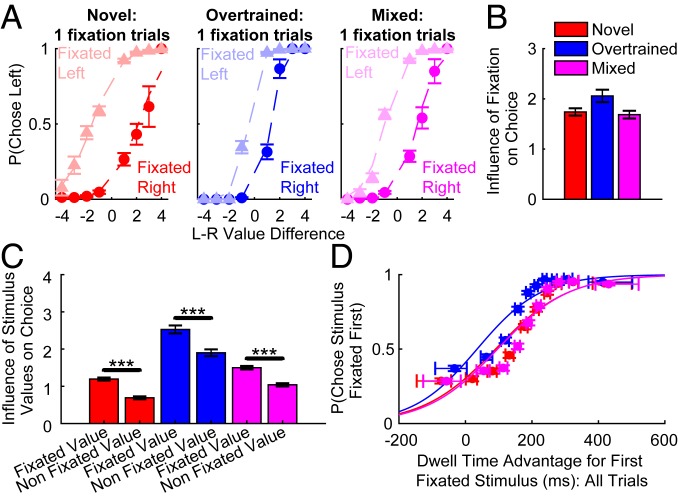
Influence of fixations on economic choice. (*A*) The influence of the direction fixated, over and above value difference, on trials where a single stimulus was fixated. A separate plot shows novel, overtrained, and mixed trials. Markers show the proportion (±SE) of left choices at a specific value difference. The lightly shaded data are for trials where subjects looked left, and the darker data for where subjects looked right. Subjects were more likely to choose the option they fixated. Dashed lines represent a logistic model fit (*SI Appendix*, *Text*). (*B*) Logistic regression coefficients (±SE) for a model predicting economic choice. The direction fixated significantly influenced choice over and above stimulus value. (*C*) The influence of fixated and nonfixated stimulus values on choices in 1-fixation trials. Logistic regression coefficients (±SE) show that both stimuli significantly influenced choices but the fixated value had a stronger influence (*SI Appendix*, *Text*). ***P < 10^−10^. (*D*) The probability of choosing the item fixated first, as a function of the final dwell time advantage allocated to that stimulus (*SI Appendix*, *Text*). The dwell times are binned into 10 deciles, with the mean choice probability (±SE) for each bin indicated with a circular marker at the median dwell time of the bin. Lines show a logistic fit of the data. Horizontal error bars show the interquartile range (i.e., central 50% values) for dwell times. This analysis includes all trials for each condition type (i.e., novel, overtrained, or mixed). Subjects were more likely to choose the first fixated item when there was a greater time advantage in fixation duration allocated to it.

As our subjects made fewer fixations than in previous human experiments (Fig. 2*A* and *SI Appendix*, Fig. S3), to explore this hypothesis we focused on the influence of fixation on trials where only a single stimulus was fixated. The proportion of left choices, as a function of left-minus-right value difference, was split into 2 psychometric functions depending upon the direction fixated (Fig. 6*A* and *SI Appendix*, Fig. S11). If fixation location had no impact on choice over and above value difference, the 2 curves in each panel would overlap. The greater the vertical offset between the 2 functions, the stronger the effect of fixation direction on choice. We found that subjects’ fixation pattern influenced choice, as they were more likely to choose a stimulus if they viewed it. This result was quantified by using a regression approach, with fixation location used to predict final choice and the effect of stimulus value controlled for by regressing this out (Fig. 6*B* and *SI Appendix*, *Text*). Model comparison further confirmed that choices were best predicted by a model incorporating the location of the subjects’ fixation, in addition to value difference (*SI Appendix*, *Text* and Table S2).

There are several possible explanations for the influence of fixation on choice we observed. In the attentional drift-diffusion model (aDDM), the subject’s momentary evidence accumulation rate is dominated by the currently fixated stimulus value. However, the value of the nonfixated stimulus still has an influence, albeit weaker (2). Alternatively, subjects could decide whether to pick the first fixated stimulus based on its value relative to the average value of all potential stimuli, without covertly processing the value of the nonfixated stimulus. We performed further regression analyses to compare the influence of the fixated and nonfixated stimulus values on choice (Fig. 6*C* and *SI Appendix*, Fig. S12). Consistent with the aDDM, the fixated stimulus value had a significantly stronger influence than the nonfixated stimulus value, but the latter still significantly influenced choice. This result reveals that choice was better explained by assuming subjects were using covert information when making choices, rather than only accumulating evidence for choice based on stimuli that were overtly fixated. Covertly evaluated stimulus values thus strongly influence both the probability of fixating those stimuli and the probability of choosing them.

To facilitate direct comparison with the human aDDM literature, we next tested the influence of fixation duration on choices with data from all trials (Fig. 6*D* and *SI Appendix*, Fig. S13). As subjects very rarely made more than 2 fixations before making a choice (*SI Appendix*, Fig. S3 *B* and *G*), they usually fixated the first stimulus for longer. Although this bias necessitates a slightly different analysis from that used in human studies, consistent with this work, the greater the time advantage for the first fixated stimulus, the more likely it was to be chosen. This finding was irrespective of the number of fixations per trial (*SI Appendix*, Fig. S13). We also examined dwell time effects after subtracting the probability of choosing the first fixated stimulus for each value difference. This controls for any possible influence of stimulus values on fixation durations, and demonstrates there remains a strong effect of total fixation time on choice (*SI Appendix*, Fig. S13).

Fixations therefore influence choice (Fig. 6) but also remain dissociable from it (Fig. 5 and *SI Appendix*, Fig. S5 vs. Fig. S6). To provide some intuition for the compatibility of these effects, they can be illustrated on trials where subjects first fixate the lower-value item. On these trials, subjects are now more likely to choose incorrectly because fixating an item first increases its chances of being selected (Fig. 6 *A* and *B* and *SI Appendix*, Fig. S14). However, economic choice is not completely determined by fixations, as subjects can covertly evaluate the other stimulus (Fig. 6*C*) or make subsequent fixations (Fig. 4) in order to optimize their final choice.

## Discussion

Here we have shown that during a free-response value-based decision-making task, subjects rapidly (within ∼150 ms) fixate valuable and novel stimuli. Subjects also showed a strong bias to initially fixate, but not to choose, novel stimuli when choosing between one novel and one overtrained stimulus. The ability to direct fixations to valuable stimuli is learned over the course of a behavioral session, whereas the novelty bias is consistent throughout. Once subjects have fixated an item, they are more likely to choose it. However, their choice is also shaped by covert evaluation of stimuli they do not explicitly view. The duration of the first fixation, and whether or not to fixate the other option, is influenced by covert evaluation of the nonfixated stimulus.

These results show that during economic choices, a covert evaluation of high-level stimulus features (value and novelty) can be used to rapidly guide fixations. Covert mechanisms also continue to bias information-sampling behavior once a stimulus is fixated, as the nonfixated stimulus value influences the likelihood of subsequently fixating it and the time spent on the first fixation. This builds upon existing studies showing that previously rewarded stimuli can draw attention, demonstrated by their slowing of visual search (12–14) and continuing to attract fixations despite being currently unrewarded (14–17). However, an important distinction is that in the context of economic choice, quickly identifying reward-predictive stimuli in our environment is evolutionarily advantageous, whereas previous studies demonstrating oculomotor capture have focused on how this phenomenon may be a signature of poor attentional control (13). As well as value, our subjects preferentially directed their fixations toward novel stimuli. This may be because subjects find it more difficult to covertly evaluate stimuli which are relatively novel, and hence it is necessary to fixate them in order to resolve uncertainty (20) and maximize momentary reward. Novel stimuli may also carry a “novelty bonus,” making them inherently more valuable in order to promote exploratory behavior and learning, consequentially optimizing reward rate across a longer timescale (20, 21). The fixation novelty bias we observed persisted across the entire behavioral session. As the covert valuation effects on first fixations in novel trials never reached the same level as in overtrained trials, it may have remained important for novel stimuli to attract fixations throughout the session.

Computational models incorporating fixation patterns have been highly successful in explaining economic choice behavior (2–4, 19). However, we are only aware of one such model which attempts to explain where subjects direct their fixations (19). Towal and colleagues (19) showed that during choices, subjects viewed stimuli which were visually salient and more valuable for longer durations. However, in their task, subjects had to view a stimulus array of 4 options for a fixed duration of 2 s before making a choice. On these trials, subjects made an average of over 5 fixations. In such cases where subjects have ample time to overtly fixate all information, it is unclear whether covert evaluation of stimuli underlies their results. Instead, subjects may simply dwell longer on (or return to) stimuli that are more salient or valuable. Our study was intended to mimic more naturalistic situations where subjects are free to fixate stimuli and choose without constraints, and hence we were able to directly investigate potential covert valuation processes influencing fixation patterns. However, it should be noted our subjects made fewer fixations compared with similar experiments performed in humans (2, 19). In our task, the visual fixations were influenced by the value of stimuli in the periphery, particularly when the values of those stimuli were well-learned. Our study therefore uniquely demonstrates that covert stimulus evaluation biases where overt attention is allocated (i.e., fixated stimulus), and ultimately what options are chosen.

Other studies in humans have reported no influence of stimulus value on first fixation direction (1–3, 6). These studies have typically used a large set of novel stimuli, with few repeated exposures to each stimulus. In these contexts, subjects usually show a fixation direction bias (i.e., to look left first regardless of stimulus values). Crucially, the amount of repeated stimulus exposure subjects receive appears to be a critical factor influencing fixation patterns. In our study, we were able to compare the effectiveness of covert evaluation processes in contexts with either relatively novel (experienced ∼10 times before data collection) or well-learned stimulus–value associations (experienced >100 times before data collection). In the first decile of data collection, first fixations were relatively random, but became significantly value-driven once subjects experienced the stimulus–outcome association ∼20 times. Our results provide insight into the time course with which subjects learn to direct fixations toward valuable stimuli, and help to reconcile why value-driven oculomotor capture has been observed extensively in some tasks but not in economic choice experiments (2, 3, 15–17). This time course resembles the acquisition of human fixation biases toward locations where the most rewarding choice options are frequently presented (22). Beyond the laboratory setting, it is important to remember that people also have strong reward associations with stimuli (e.g., favorite snack food), so it is plausible the covert valuation mechanism we observed would be involved in guiding our decisions in many contexts throughout the day.

Our results also show that the decision processes for directing fixations and economic choices are dissociable. If both of these decisions were considered as evidence accumulation processes (2, 19), it is feasible that the same accumulator could be responsible for both, with a lower evidence threshold required for gaze shifts. Here, we provide several key pieces of evidence against this hypothesis. First, the time course over which the 2 types of decision are learned is different. Subjects rapidly learn to make accurate economic choices between reward-predictive stimuli after a short period of secondary conditioning. However, the value-driven fixations become increasingly more common as stimuli become more familiar. Furthermore, fixations, but not choices, were strongly biased by novelty on mixed trials. Finally, the reaction time for economic choices is influenced by the relative values of the 2 options, consistent with an evidence accumulation process. However, the first fixation latency was inconsistently influenced by stimulus values, meaning this covert evaluation does not clearly resemble an evidence accumulation process. Together, this suggests separate decision processes for guiding fixations and economic choices. Our findings resemble earlier work showing that even though the value of novel cues can be quickly learned, they continue to attract attention (23).

Given these clear dissociations, it is important to consider if different brain regions underlie fixation direction and economic choices. Evidence suggests that the prefrontal cortex (PFC) is critical for value-based decision making (24–26), and PFC neurons encode the values of choice options (27, 28). Relevant to our results, recent studies show that value coding of neurons in the orbitofrontal cortex, and the value difference BOLD signal in human fMRI experiments, is modulated by attention (29–32). However, the latency of value encoding in PFC neurons typically exceeds 200 ms (27, 31, 33). Yet, in the current study, we identified value-guided first fixations within 150 ms of stimulus presentation. Taken together, it seems unlikely PFC activity underscores this short-latency covert evaluation process that directs fixations to more valuable and salient (novel) information.

Instead, the subcortical saccadic system—composed of the caudate nucleus, substantia nigra pars reticulata, and superior colliculus (34)—is a strong candidate for several reasons. Neurons within these brain regions rapidly receive cortical input, and have been shown to discriminate the value of stimuli within ∼100 ms of their onset (15, 16, 35). Stimulus value signals encoded by the caudate nucleus are relayed to the superior colliculus via the substantia nigra (36), to bias fixations to more valuable information. Relevant to our findings, one model suggests the head of the caudate influences fixations based on flexible, recently updated values, whereas the caudate tail circuit relies on long-term memories to automatically bias fixations to valuable well-learned stimuli (37). Future investigations could test if the neural value representations for novel and overtrained stimuli are also segregated within the basal ganglia, and how this relationship develops through learning. Aside from the subcortical saccadic system, the superior colliculus also receives direct input from cortical visual areas (34), and thus higher visual areas (such as V4) may be involved in the valuation process (38) which biases these fast first fixations. Our findings challenge current models suggesting economic choice is exclusively the domain of the PFC (39), instead suggesting valuation processes also occur in other brain structures, potentially before—or in parallel with—the PFC (40). An important avenue for future research in information seeking and decision making is to better understand the interplay and competition between this fast covert valuation system, which biases gaze in ∼150 ms, and the slower PFC valuation system.

In summary, our study reconciles 2 literatures which appeared to make diametrically opposite predictions. Until now, it remained completely unknown why stimulus value influenced fixation deployment in one set of experiments (15, 17) but had no influence in other experiments (1–3). We demonstrated that the impact of value on fixations depends upon whether that value is well-learned or has only recently been learned. The covert valuation processes we observed biased fixations within 150 ms, and influenced what subjects chose. It is therefore important for future models of the neural and computational bases of information search and decision making to account for the prominent role of covert evaluations our study has highlighted.

## Methods

Two adult male rhesus monkeys (*Macaca mulatta*), M and F, were used as subjects in the study. They weighed 7 to 10 kg at the time of data collection. Daily fluid intake was regulated in order to maintain subject motivation. All experimental procedures were approved by the University College London Animal Welfare and Ethical Review Body (AWERB), and carried out in accordance with the UK Animals (Scientific Procedures) Act. A detailed description of the behavioral task and analysis methods is provided in *SI Appendix*.

## Supplementary Material

Supplementary File

## References

[1] Cavanagh J. F., Wiecki T. V., Kochar A., Frank M. J., Eye tracking and pupillometry are indicators of dissociable latent decision processes. J. Exp. Psychol. Gen. 143, 1476–1488 (2014). 24548281 10.1037/a0035813 PMC4114997

[2] Krajbich I., Armel C., Rangel A., Visual fixations and the computation and comparison of value in simple choice. Nat. Neurosci. 13, 1292–1298 (2010). 20835253 10.1038/nn.2635

[3] Krajbich I., Rangel A., Multialternative drift-diffusion model predicts the relationship between visual fixations and choice in value-based decisions. Proc. Natl. Acad. Sci. U.S.A. 108, 13852–13857 (2011). 21808009 10.1073/pnas.1101328108 PMC3158210

[4] Krajbich I., Accounting for attention in sequential sampling models of decision making. Curr. Opin. Psychol. 29, 6–11 (2018). 30368108 10.1016/j.copsyc.2018.10.008

[5] Armel K. C., Beaumel A., Rangel A., Biasing simple choices by manipulating relative visual attention. Judgm. Decis. Mak. 3, 396–403 (2008).

[6] Reutskaja E., Nagel R., Camerer C. F., Rangel A., Search dynamics in consumer choice under time pressure: An eye-tracking study. Am. Econ. Rev. 101, 900–926 (2011).

[7] Theeuwes J., Perceptual selectivity for color and form. Percept. Psychophys. 51, 599–606 (1992). 1620571 10.3758/bf03211656

[8] Nothdurft H. C., Attention shifts to salient targets. Vision Res. 42, 1287–1306 (2002). 12044759 10.1016/s0042-6989(02)00016-0

[9] Itti L., Koch C., Computational modelling of visual attention. Nat. Rev. Neurosci. 2, 194–203 (2001). 11256080 10.1038/35058500

[10] van Zoest W., Donk M., Theeuwes J., The role of stimulus-driven and goal-driven control in saccadic visual selection. J. Exp. Psychol. Hum. Percept. Perform. 30, 746–759 (2004). 15305440 10.1037/0096-1523.30.4.749

[11] Theeuwes J., Kramer A. F., Hahn S., Irwin D. E., Zelinsky G. J., Influence of attentional capture on oculomotor control. J. Exp. Psychol. Hum. Percept. Perform. 25, 1595–1608 (1999). 10641312 10.1037//0096-1523.25.6.1595

[12] Theeuwes J., Belopolsky A. V., Reward grabs the eye: Oculomotor capture by rewarding stimuli. Vision Res. 74, 80–85 (2012). 22902641 10.1016/j.visres.2012.07.024

[13] Anderson B. A., Laurent P. A., Yantis S., Value-driven attentional capture. Proc. Natl. Acad. Sci. U.S.A. 108, 10367–10371 (2011). 21646524 10.1073/pnas.1104047108 PMC3121816

[14] Anderson B. A., Yantis S., Value-driven attentional and oculomotor capture during goal-directed, unconstrained viewing. Atten. Percept. Psychophys. 74, 1644–1653 (2012). 22810561 10.3758/s13414-012-0348-2 PMC3499680

[15] Yasuda M., Yamamoto S., Hikosaka O., Robust representation of stable object values in the oculomotor basal ganglia. J. Neurosci. 32, 16917–16932 (2012). 23175843 10.1523/JNEUROSCI.3438-12.2012 PMC3537824

[16] Kim H. F., Hikosaka O., Distinct basal ganglia circuits controlling behaviors guided by flexible and stable values. Neuron 79, 1001–1010 (2013). 23954031 10.1016/j.neuron.2013.06.044 PMC3782315

[17] Ghazizadeh A., Griggs W., Hikosaka O., Ecological origins of object salience: Reward, uncertainty, aversiveness, and novelty. Front. Neurosci. 10, 378 (2016). 27594825 10.3389/fnins.2016.00378 PMC4990562

[18] Lohse G. L., Consumer eye movement patterns on yellow pages advertising. J. Advert. 26, 61–73 (1997).

[19] Towal R. B., Mormann M., Koch C., Simultaneous modeling of visual saliency and value computation improves predictions of economic choice. Proc. Natl. Acad. Sci. U.S.A. 110, E3858–E3867 (2013). 24019496 10.1073/pnas.1304429110 PMC3791693

[20] Dayan P., Daw N. D., Decision theory, reinforcement learning, and the brain. Cogn. Affect. Behav. Neurosci. 8, 429–453 (2008). 19033240 10.3758/CABN.8.4.429

[21] Kakade S., Dayan P., Dopamine: Generalization and bonuses. Neural Netw. 15, 549–559 (2002). 12371511 10.1016/s0893-6080(02)00048-5

[22] Colas J. T., Lu J., Learning where to look for high value improves decision making asymmetrically. Front. Psychol. 8, 2000 (2017). 29187831 10.3389/fpsyg.2017.02000 PMC5695242

[23] Foley N. C., Jangraw D. C., Peck C., Gottlieb J., Novelty enhances visual salience independently of reward in the parietal lobe. J. Neurosci. 34, 7947–7957 (2014). 24899716 10.1523/JNEUROSCI.4171-13.2014 PMC4044252

[24] Gläscher J., Lesion mapping of cognitive control and value-based decision making in the prefrontal cortex. Proc. Natl. Acad. Sci. U.S.A. 109, 14681–14686 (2012). 22908286 10.1073/pnas.1206608109 PMC3437894

[25] Kennerley S. W., Walton M. E., Behrens T. E., Buckley M. J., Rushworth M. F., Optimal decision making and the anterior cingulate cortex. Nat. Neurosci. 9, 940–947 (2006). 16783368 10.1038/nn1724

[26] Rudebeck P. H., Frontal cortex subregions play distinct roles in choices between actions and stimuli. J. Neurosci. 28, 13775–13785 (2008). 19091968 10.1523/JNEUROSCI.3541-08.2008 PMC6671924

[27] Kennerley S. W., Dahmubed A. F., Lara A. H., Wallis J. D., Neurons in the frontal lobe encode the value of multiple decision variables. J. Cogn. Neurosci. 21, 1162–1178 (2009). 18752411 10.1162/jocn.2009.21100 PMC2715848

[28] Padoa-Schioppa C., Assad J. A., Neurons in the orbitofrontal cortex encode economic value. Nature 441, 223–226 (2006). 16633341 10.1038/nature04676 PMC2630027

[29] McGinty V. B., Rangel A., Newsome W. T., Orbitofrontal cortex value signals depend on fixation location during free viewing. Neuron 90, 1299–1311 (2016). 27263972 10.1016/j.neuron.2016.04.045 PMC4911340

[30] Xie Y., Nie C., Yang T., Covert shift of attention modulates the value encoding in the orbitofrontal cortex. eLife 7, e31507 (2018). 29533184 10.7554/eLife.31507 PMC5871329

[31] Hunt L. T., Triple dissociation of attention and decision computations across prefrontal cortex. Nat. Neurosci. 21, 1471–1481 (2018). 30258238 10.1038/s41593-018-0239-5 PMC6331040

[32] Lim S. L., O’Doherty J. P., Rangel A., The decision value computations in the vmPFC and striatum use a relative value code that is guided by visual attention. J. Neurosci. 31, 13214–13223 (2011). 21917804 10.1523/JNEUROSCI.1246-11.2011 PMC6623246

[33] Cavanagh S. E., Wallis J. D., Kennerley S. W., Hunt L. T., Autocorrelation structure at rest predicts value correlates of single neurons during reward-guided choice. eLife 5, e18937 (2016). 27705742 10.7554/eLife.18937 PMC5052031

[34] Hikosaka O., Takikawa Y., Kawagoe R., Role of the basal ganglia in the control of purposive saccadic eye movements. Physiol. Rev. 80, 953–978 (2000). 10893428 10.1152/physrev.2000.80.3.953

[35] Griggs W. S., Amita H., Gopal A., Hikosaka O., Visual neurons in the superior colliculus discriminate many objects by their historical values. Front. Neurosci. 12, 396 (2018). 29942248 10.3389/fnins.2018.00396 PMC6004417

[36] Yasuda M., Hikosaka O., Functional territories in primate substantia nigra pars reticulata separately signaling stable and flexible values. J. Neurophysiol. 113, 1681–1696 (2015). 25540224 10.1152/jn.00674.2014 PMC4359992

[37] Hikosaka O., Kim H. F., Yasuda M., Yamamoto S., Basal ganglia circuits for reward value-guided behavior. Annu. Rev. Neurosci. 37, 289–306 (2014). 25032497 10.1146/annurev-neuro-071013-013924 PMC4148825

[38] Baruni J. K., Lau B., Salzman C. D., Reward expectation differentially modulates attentional behavior and activity in visual area V4. Nat. Neurosci. 18, 1656–1663 (2015). 26479590 10.1038/nn.4141 PMC4624579

[39] Padoa-Schioppa C., Neurobiology of economic choice: A good-based model. Annu. Rev. Neurosci. 34, 333–359 (2011). 21456961 10.1146/annurev-neuro-061010-113648 PMC3273993

[40] Hunt L. T., Hayden B. Y., A distributed, hierarchical and recurrent framework for reward-based choice. Nat. Rev. Neurosci. 18, 172–182 (2017). 28209978 10.1038/nrn.2017.7 PMC5621622

