# Supplementary material for: Visual fixation patterns during economic choice reflect covert valuation processes that emerge with learning
Source: Proc Natl Acad Sci U S A. 2019 Oct 21;116(45):22795–801. doi: 10.1073/pnas.1906662116 (PMC6842638; doi:10.1073/pnas.1906662116)
Supplement: Supplementary File [file pnas.1906662116.sapp.pdf]

PNAS
www.pnas.org

Supplementary Information for

Visual fixation patterns during economic choice reflect covert valuation processes that emerge with learning

Sean E. Cavanagh, W.M. Nishantha Malalasekera, Bruno Miranda, Laurence T. Hunt, and Steven W. Kennerley

Sean E. Cavanagh, Steven W. Kennerley
Email: sean.cavanagh.12@ucl.ac.uk, s.kennerley@ucl.ac.uk

This PDF file includes:

Supplementary text
Figures S1 to S14
Tables S1 to S3
SI References

www.pnas.org/cgi/doi/10.1073/pnas.1906662116

Supplementary Information Text

SI Methods

Behavioural Task

Details of the experimental setup have been described in detail previously (1). The behavioural paradigm was run using the MATLAB based toolbox MonkeyLogic (http://www.monkeylogic.net/, Brown University, USA) (2-4). A photodiode test was performed to benchmark the system, confirming eventmarkers precisely indicated the time of task events (within 2ms). We monitored eye position and pupil dilation during the task using an infra-red system (ISCAN ETL-200) sampling at 240Hz. Monkeys used a joystick to report their economic choices. All joystick and eye position data was relayed to MonkeyLogic for use online during the task. It was also interpolated, and recorded by MonkeyLogic at 1000Hz for offline analysis.

Subjects performed a value-based decision-making task (Choice Phase; **Fig. 1A**) following a short conditioning phase. In the conditioning phase (**SI Appendix: Fig. S1**), subjects learned the values of 10 Novel reward-predictive pictorial stimuli. Half of the stimuli indicated the probability of receiving reward (10%, 30%, 50%, 70%, 90%) and the other half were associated with one of five magnitudes of reward size (0.14g, 0.33g, 0.51g, 0.71g, 0.90g). The secondary conditioning procedure consisted of one-alternative ‘forced choice’ trials for subjects to learn the stimulus values. In a single block of trials, subjects completed a ‘forced-choice’ trial for each value of a particular attribute (probability or magnitude), then attempted two choice trials (**SI Appendix: Fig. S1A**) between two of the stimuli. Blocks alternated between attributes. Ten blocks were completed for each attribute.

Following completion of the conditioning phase, the choice phase began. In the choice phase, only two-alternative choice trials were presented (i.e., no ‘forced-choice’ trials presented). In addition to the Novel stimuli subjects had just learned, 10 other reward-predictive stimuli were also presented as choice options. Subjects had been heavily exposed to these additional stimuli in previous training sessions prior to the first data collection session (M: ~1500, F: ~3000 total exposures to the stimulus set), hence they were referred to as Overtrained. The same 10 Overtrained stimuli were used in each behavioural session. Novel stimuli were not reused as Overtrained stimuli in subsequent behavioural sessions.

Subjects could be given trials consisting of both Overtrained stimuli (Overtrained trials; **Fig. 1C**), two Novel stimuli (Novel trial) or one of each (Mixed trials). Subjects were always asked to make choices within a certain attribute (e.g. choosing between probabilities) and never between attributes. Subjects never had to choose between two stimuli of equal value – therefore optimality was

defined as choosing the most valuable option. All trial types were pseudorandomly interleaved.

A representation of a choice trial timeline can be found in **Fig. 1B**. Subjects initiated each trial by returning the joystick to its centre position. At this point, a white background appeared on the screen with a red central fixation square (0.5 x 0.5 visual degrees in size). Subjects were required to fixate the red square for a continuous 500ms (fixation radius of 3 visual degrees) within a 10s time period. If this was not achieved, a short 'timeout' was given and the trial restarted. Once the fixation period was completed, the fixation spot disappeared and two isoluminant picture stimuli (100 x 100 pixels) were presented 6.5 visual degrees to the left and right of the centre. Importantly, subjects were free to saccade to anywhere on (or off) the screen and to choose a stimulus using a left/right joystick response at any time after the stimuli were presented. If subjects did not respond within 5s the trial was aborted. Once the response was made, a grey square was drawn around the chosen stimulus and a 500ms pre-feedback period was initiated. After the pre-feedback period, the unchosen stimulus was removed from the screen and the reward epoch was initiated. Subjects were rewarded with juice (according to the reward probabilities and volumes described above) delivered to the mouth using a precise peristaltic pump (ISMATEC IPC).

A representation of a conditioning trial timeline can be found in **SI Appendix: Fig. S1C**. In these trials, following successful fixation, a single stimulus appeared on either the left or right of the screen. Once the stimulus was chosen by the joystick response, it again remained highlighted for 500ms. However, following this initial highlighted period, the stimulus then disappeared from the screen. After a further 500ms delay, a secondary reinforcer appeared for 500ms. This was a coloured bar on top of a white background. The height of the bar indicated the chosen stimulus value. After the secondary reinforcer disappeared, there was a prefeedback period before the reward epoch. Data from this conditioning phase is not described in the current report.

After completing a behavioural session of this task on 'Day 1', subjects then performed a different decision-making task on subsequent sessions ('Days 2-4') using the Novel stimuli learned on 'Day 1'. This testing schedule then restarted with a new 'Day 1' session. Only data from these 'Day 1' sessions is described in the present report.

Behavioural Analysis

For each trial, eye position data was analysed from the time of stimuli onset until the joystick was moved outside of a central two-degree visual radius. This time period was defined as the subject's reaction time for the trial (**Fig. 1E, SI Appendix: Fig. S2**).

In order to determine what information the subject fixated on the screen, a region of interest (ROI) was defined for each stimulus. At any given time, subjects were considered to be viewing a stimulus if the recorded x-coordinate of the eye position data was within 2.5 degrees of the centre of the stimulus. The number of stimuli viewed per trial (**Fig. 2A**) was calculated by determining if one or both stimuli were viewed for at least 15ms. The number of fixations in each trial (**SI Appendix: Fig. S3B, G**) summed the number of distinct 15ms periods that stimuli were fixated. To be considered a separate fixation, subjects had to switch their gaze between the two stimuli. For example, if their eye position were inside the left stimulus ROI for 200ms, then in neither ROI for 5ms, then returned to the left stimulus ROI for a further 100ms, this would only be considered a single fixation (as opposed to two separate fixations).

The latency of the first fixation (**Fig. 2B**), and which stimulus was fixated first, were defined using a saccade detection algorithm (5, 6). Eye position data was zero-phase filtered using a second order butterworth filter with a cut off frequency of 35Hz. A threshold of 7 degrees/second, horizontal distance of greater than 4 degrees, and minimum duration of 20ms were used. For each trial, the first detected saccade defined the first fixation latency and direction. The first stimulus dwell time (**Fig. 4, SI Appendix: Fig. S8**) was determined to be the viewing time allocated to the stimulus initially fixated. The dwell time advantage for the first fixated stimulus (**Fig. 6, SI Appendix: Fig. S13**) was the first stimulus dwell time minus the total viewing time allocated to the other stimulus. On trials where only a single stimulus was fixated, the total viewing time allocated to the other stimulus was 0ms.

On a small proportion of completed trials (0.47%) no saccades were detected using the saccade detection algorithm. If the ROI analysis described above indicated the subject had fixated a stimulus for >15ms on these trials, this suggested either a saccade had occurred below the algorithm's thresholds, or the subject had not moved his gaze towards one of the stimuli with a single ballistic eye movement. On these trials, the first fixation direction was defined based upon the first ROI acquired. Fixation latency was left undefined.

The majority of data analysis utilised logistic regression and was performed using data collapsed across all sessions for a given subject, unless otherwise stated (correlation across session analyses in **Fig. 3A, SI Appendix: Fig. S5, Fig. S6, Fig. S10**). Logistic regressions were performed using Equation 1 where YP is the probability of observing an event, b_0 is a constant term, b_n is a weighting coefficient and x_n is a regressor:

$$\boldsymbol{YP} = \frac{\mathbf{1}}{\mathbf{1} + \boldsymbol{e}^{-(\boldsymbol{b_0}+\boldsymbol{b_1 x_1}+\boldsymbol{b_2 x_2}+\cdots+\boldsymbol{b_n x_n})}} \qquad \text{Equation 1}$$

All linear regressions were performed using Equation 2 where Y is the dependant variable, b_0 is a constant term, b_n is a weighting coefficient and x_n is a regressor:

$$\boldsymbol{Y = b_0 + b_1 X_1 + b_2 X_2 + \cdots + b_n X_n} \qquad \text{Equation 2}$$

SI Appendix: Table S3 contains detailed descriptions of the regression models used to analyse behaviour. Comparisons between relevant regressors were performed using a linear hypothesis test. As the first stimulus dwell time (**Fig. 4D**), reaction time (**SI Appendix: Fig. S2C, G**), and latency of first fixation (**SI Appendix: Fig. S3E, J**) variables were not normally distributed, they were log-transformed before performing linear regression analysis. The dwell time data was further z-scored to make for a clearer visualisation (**Fig. 4C**), but the log-transformed variable was used in the relevant analyses.

In order to test how fixation behaviour changed over the course of a behavioural session, we used a logistic regression approach (**Fig. 3B, SI Appendix: Fig. S5, Fig. S6, Fig. S10**). The data from each behavioural session was subdivided into deciles based upon the trial number for the relevant trial type (e.g. Novel trials in **Fig. 3B, SI Appendix: Fig. S5, Fig. S6;** Mixed trials in **SI Appendix: Fig. S10**). This data for each trial decile was then pooled across sessions. When performing the regression analyses, there was a separate constant term and value difference term for each decile (**SI Appendix: Table S3**). Therefore, we could assess if fixations became more value driven by comparing the 10 value-difference regression coefficients (**Fig. 3B, SI Appendix: Fig. S5, Fig. S6**). We could also evaluate whether the novelty bias changed over the course of a session by reviewing the 10 constant term regression coefficients (**SI Appendix: Fig. S10**). This analysis is therefore complementary to testing whether the proportion of fixations to the more valuable (**Fig. 3A**) or Novel stimulus (**SI Appendix: Fig. S10A**) change over the session. The regression analysis additionally isolates any value-based effects from bias effects. When assessing if fixations became more driven by value on Novel trials (**Fig. 3B**), this approach would be useful to control for subjects potentially showing a strong direction bias which diminished across the course of a session. It is particularly important for testing any changes in the novelty bias (**SI Appendix: Fig. S10**), as we needed to additionally control for fixations becoming more value driven on Mixed trials as the Novel stimulus value became well-learned.

To test the effects of fixation pattern on economic choice, over and above the effects of value, we used a regression approach (**Fig. 6A-B; SI Appendix: Table S2**). We initially fitted data from all trials where subjects fixated a single stimulus, using a model with 9 regressors (**SI Appendix: Table S2,** Model 1; **SI Appendix: Table S3,** Full model). In this model there were three predictors for each trial type: a constant term, left minus right value difference, and the direction fixated. By including value difference as a co-regressor, we could study any additional effects of fixation pattern (**Fig. 6B**).

To confirm the direction fixated had an impact on choice, a cross-validation procedure was subsequently used to compare between regression models when the relevant three predictors were removed (**SI Appendix: Table S2**). We achieved this by first estimating model parameters by performing a logistic regression to predict left choice on a random half of the trials. The remaining half of the trials were used to compute model evidence (**SI Appendix: Table S2**). This process was repeated with 10000 splits of the trials, and the average log-likelihood of each model is reported. The Bayesian Information Criterion, which calculates model evidence with a penalty for additional parameters, is also reported for when the model is fitted to all the available data.

Several figure panels present distributions of latency measures (i.e. first fixation **Fig. 2B, SI Appendix: Fig. S3D, I**; reaction time **SI Appendix: Fig. S2D, H**). Some extreme outliers are left outside of the axes limits for visualisation purposes. However, these trials were included for all statistical analyses. The number of points outside the axes limits is listed in the legends of the relevant supplementary figure.

Data availability

Behavioural data and custom code for recreating the analyses will be available from the corresponding authors on request.

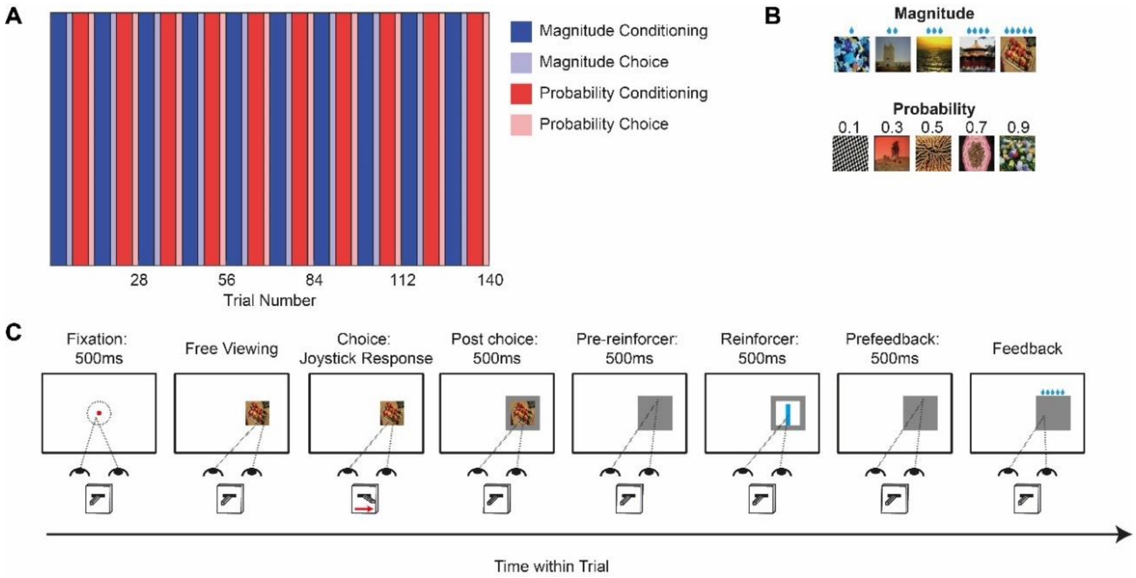

Fig. S1 Secondary conditioning for learning Novel stimulus values

Subjects began each session with the 'Conditioning Phase' where they were given 10 one-alternative forced-choice trials of 10 Novel stimuli (100 trials in total) in order to learn their values. Five of the stimuli indicated the probability of receiving reward (10%, 30%, 50%, 70%, 90%) and the other five were associated with one of five magnitudes of reward (0.14g, 0.33g, 0.51g, 0.71g, 0.90g). Secondary conditioning (with a pre-learned bar stimulus) was used to aid learning in these trials. 40 two-alternative choice trials were periodically interleaved between the one-alternative forced-choice trials. (A) Conditioning Phase Structure. Subjects completed 10 blocks of 7 trials for each attribute (magnitude and probability). Each block consisted of a one-alternative forced choice trial for each of the 5 stimuli of an attribute, then two choice trials from this attribute. The block alternated between magnitude and probability trials, with the first being randomly determined (in this schematic it is magnitude). (B) Example of a Novel stimulus set learned during an experimental session. Each stimulus is associated with a reward magnitude (top row) or a reward probability (bottom row). (C) Task Diagram for an example one-alternative forced choice magnitude trial. Subjects initiated the task by fixating on a central red fixation point for 500ms after which one pseudorandomly chosen cue was presented on either the left or the right of the screen. Subjects were free to saccade around the screen and make a manual joystick response at any time. If the joystick was moved in the direction of the stimulus, the cue was highlighted with a grey border. After 500ms, the stimulus was removed from the screen. After a further 500ms delay, a secondary reinforcer appeared. The height of this bar indicated the value of the chosen stimulus, and the colour of the bar indicated the attribute (blue bar: magnitude, black bar: probability attribute). The secondary reinforcer was removed after 500ms. After a prefeedback delay, reward was delivered.

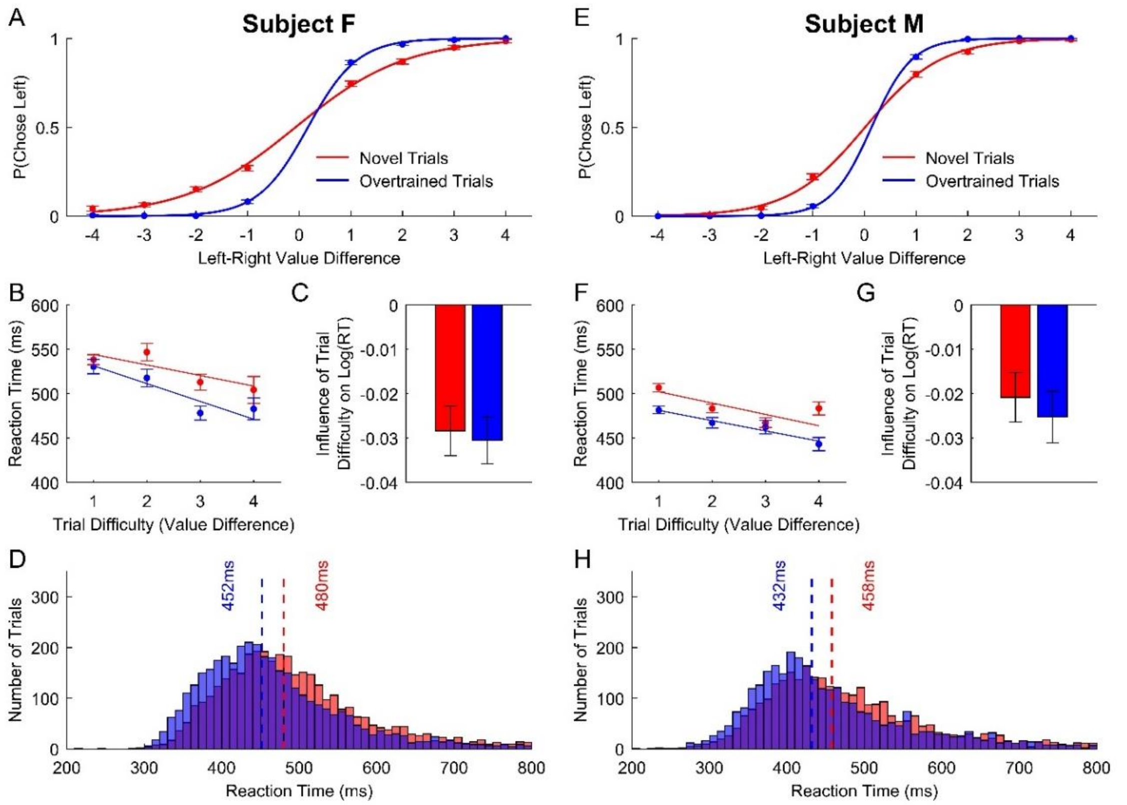

Fig. S2 Subjects' choice performance and reaction time.

(A) Choice accuracy was a function of the value difference between the stimuli. Lines show a Logistic fit of the probability of choosing the left stimulus as a function of the value difference between the left and right stimuli (Subject F: $T(7723)_{(Novel)}$=34.51, $T(7723)_{(Overtrained)}$=31.30, Subject M: $T(6297)_{(Novel)}$=31.45, $T(6297)_{(Overtrained)}$=26.12, $p<10^{-10}$ for all comparisons). (B) Reaction time was a function of trial difficulty: the absolute value difference between the two stimuli. Subjects made decisions more quickly on easier trials. Lines show a linear regression fit to the raw reaction time data. (C) Linear regression coefficients of logged reaction time as a function of the trial difficulty (Subject F: $T(3858)_{(Novel)}$= -5.073, $T(3865)_{(Overtrained)}$= -5.751; Subject M: $T(3125)_{(Novel)}$= -3.747, $T(3172)_{(Overtrained)}$= -4.348, $p<10^{-3}$ for all comparisons). (D) Histograms of the subjects' reaction times in a Novel (red) and Overtrained (blue) trial. Dashed vertical lines show the median for each trial type. Subjects respond significantly faster on Overtrained trials (Mann-Whitney U-test, $p<10^{-10}$ for both subjects). Some outliers (Subject F: 12 trials<200ms, 413 trials>800ms; Subject M, 15 trials<200ms, 177 trials>800ms) are outside of the axes limits for visualisation purposes, but were included in the analyses. All errorbars show the standard error. (E-H) As in (A-D), except for Subject M.

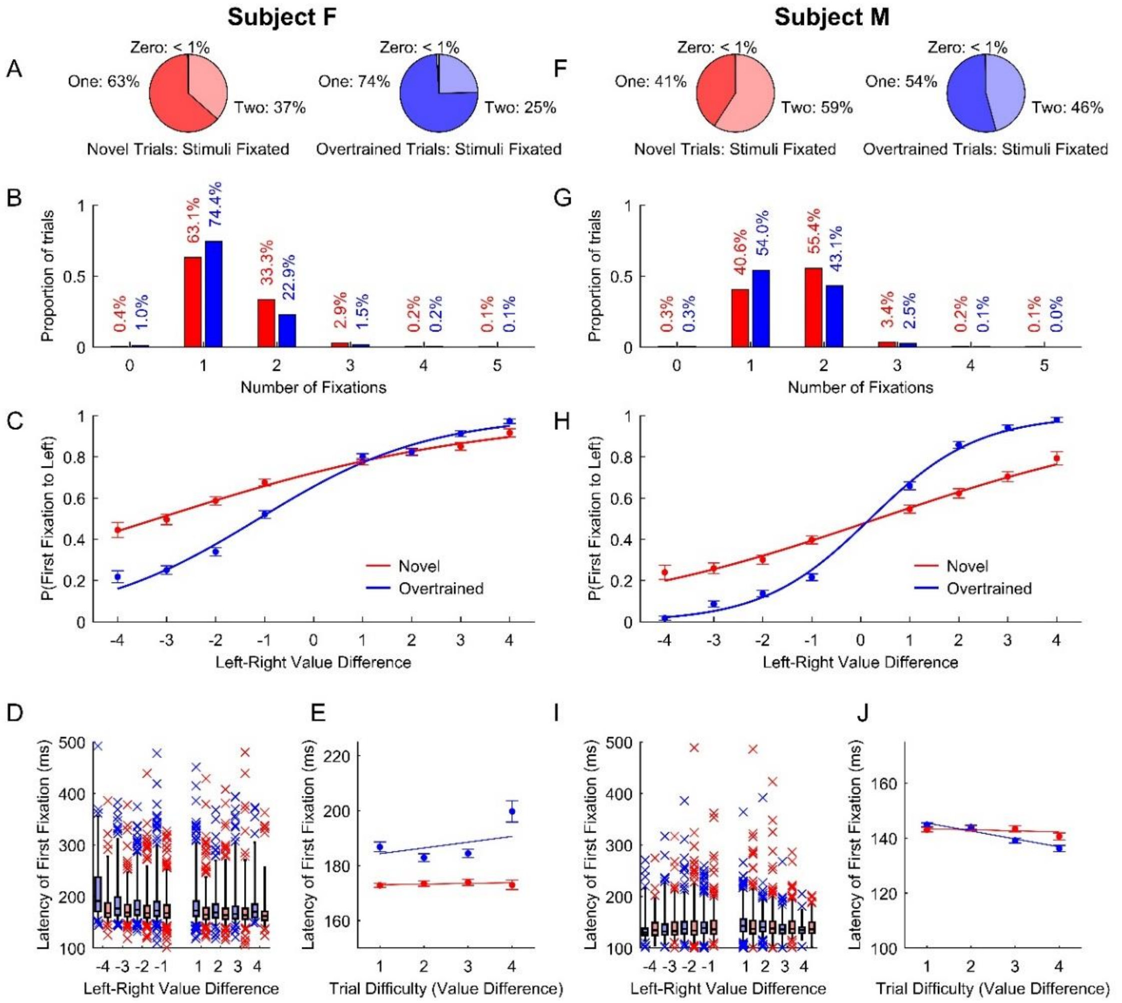

Fig. S3 Influence of value on first fixation direction and latency

(A) The proportion of trials where each subject fixated neither, one or both stimuli. (B) The proportion of trials with different numbers of total fixations. Subjects rarely make more than two fixations in a single trial. This shows that when subjects fixate both stimuli (see A), they rarely return to the initially fixated stimulus. (C) The probability of fixating the left stimulus first as a function of the value difference between the left and right stimuli. Data is separated for Novel (red) and Overtrained (blue) trials. Both subjects are more likely to direct their initial fixation to more valuable stimuli. This effect is stronger on Overtrained trials than on Novel trials. Errorbars represent the standard error. Lines show a Logistic fit of the probability of fixating the left stimulus first as a function of the value difference between the left and right stimuli (Subject F: $T(7679)_{(Novel)}$=17.10, $T(7679)_{(Overtrained)}$=28.39, Subject M: $T(6281)_{(Novel)}$=17.86, $T(6281)_{(Overtrained)}$=31.03, $p<10^{-10}$ for all comparisons).(D) Boxplots show the distribution of first fixation latencies for each subject. Each value difference has a pair of boxplots; the left-side for Overtrained trials (blue), and the right-side for Novel trials (red). The area contained within the whiskers of the boxplots represents the 95th percentile range. The box limits represent the upper and lower quartiles of the distribution. The central mark is the median of the distribution. Some extreme outliers (Subject F: 27 trials<100ms, 13 trials>500ms; Subject M, 87 trials<100ms, 1 trial>500ms) are outside of the axes limits for visualisation purposes. However, these trials were included for all statistical analyses. All trials are included, regardless of choice accuracy or the direction of the initial fixation. (E) Latency of first fixation as a function of trial difficulty: the absolute value difference between the two stimuli. The latency of this fixation is not consistently influenced by value across trial types and subjects. Lines show a linear regression fit to the raw data. Regression coefficients were subsequently calculated using log-transformed latency data (Subject F: $T(3845)_{(Novel)}$= 0.4617, $T(3829)_{(Overtrained)}$= 2.827; $p_{(Novel)}$= 0.6444, $p_{(Overtrained)}$= 0.004726; Subject M: $T(3111)_{(Novel)}$= 0.04346, $T(3159)_{(Overtrained)}$= -6.372; $p_{(Novel)}$= 0.9653, $p_{(Overtrained)}$= 2.133×10^{-10}). (F-J) As in (A-E), except for Subject M.

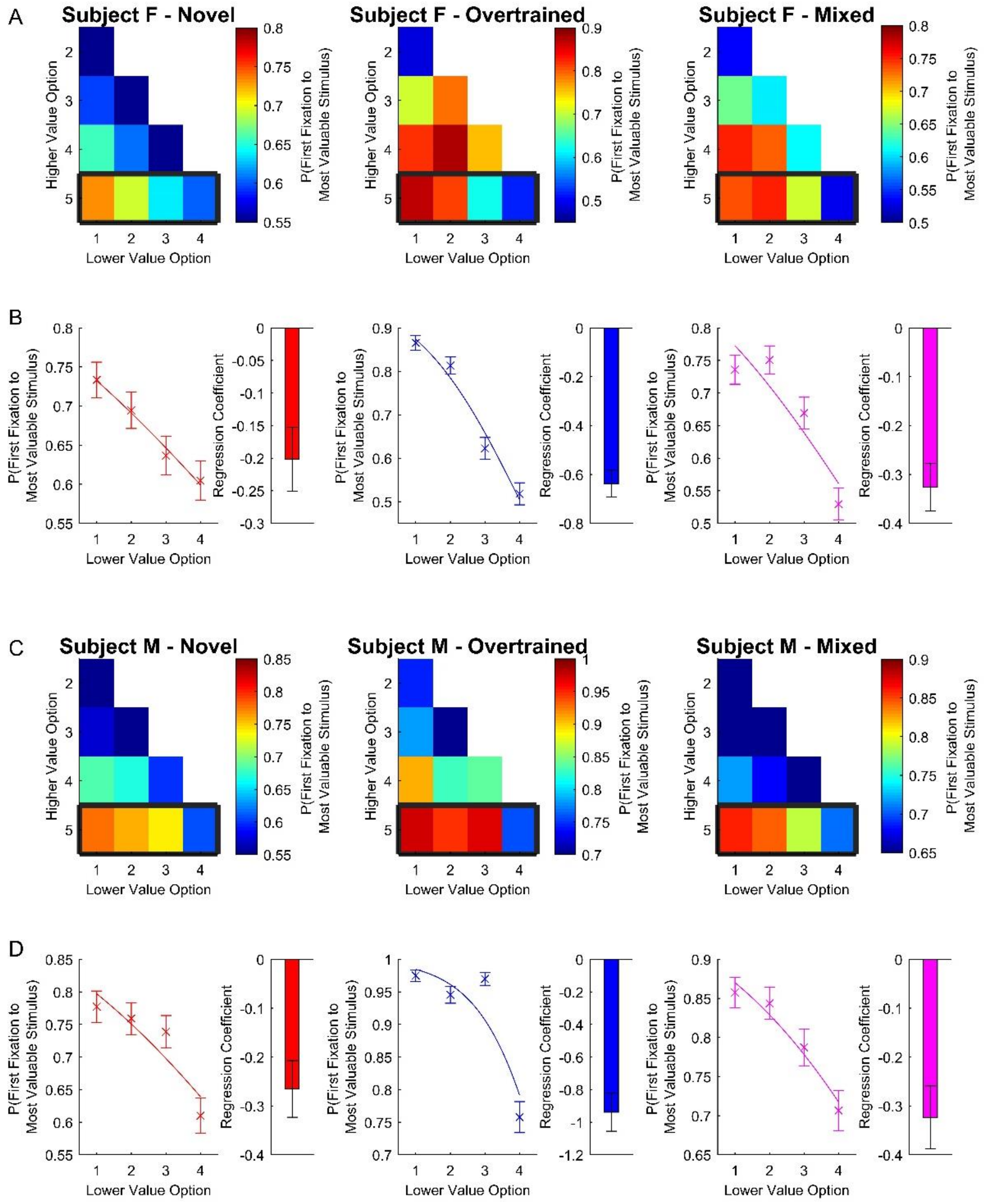

Fig. S4 Subjects' probability of fixating the best stimulus first is influenced by the lower value option, even when the highest value stimulus is available

A) The proportion of trials where Subject F fixated the most valuable stimulus first, as a function of the stimulus values. There is a separate heatmap for Novel, Overtrained and Mixed trials. B) The line graphs show the likelihood (±S.E) the most valuable stimulus is fixated first, specifically on trials where the best possible stimulus is available (i.e. Higher value option = 5, black box in panel A), for each trial type. The bar charts show the regression coefficient (±S.E) when the lower value option is used to predict the probability of fixating the best stimulus first on these trials (Subject F: $T(4661)_{(Novel)}= -4.118$, $T(4661)_{(Overtrained)}= -11.48$, $T(4661)_{(Mixed)}= -6.639$; $p_{(Novel)}= 3.821 \times 10^{-5}$, $p_{(Overtrained)} < 10^{-10}$, $p_{(Mixed)} < 10^{-10}$; Subject M: $T(3742)_{(Novel)}= -4.579$, $T(3742)_{(Overtrained)}= -8.055$, $T(3742)_{(Mixed)}= -5.022$; $p_{(Novel)}= 4.662 \times 10^{-6}$, $p_{(Overtrained)} < 10^{-10}$, $p_{(Mixed)}= 5.114 \times 10^{-7}$). C-D) As in panels A-B, for Subject M.

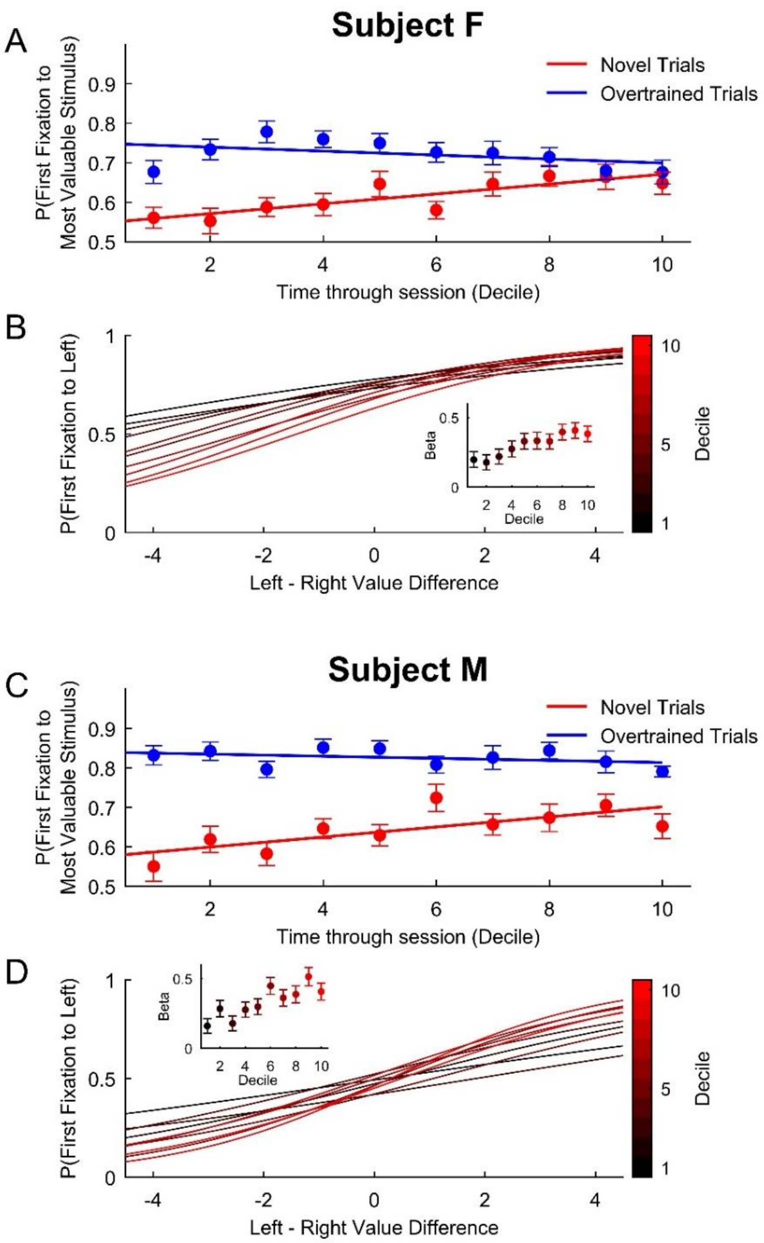

Fig. S5 The influence of stimulus value on first fixation direction across a behavioural session

On Novel trials early in a session, subjects' first fixation direction is not strongly influenced by value. However, as the stimuli become more familiar, the subjects develop a preference to fixate the more valuable stimulus first. (A) The mean (±SEM) proportion of trials where the subjects' first fixation was towards the more valuable stimulus across sessions. (A) Subject F showed a significant increase across the behavioural session for Novel trials (Spearman Correlation, r = 0.2511, p = 3.354×10^{-4}), but no change for Overtrained trials (Spearman Correlation, r = -0.0989, p = 0.1637). There was a significant difference between Novel and Overtrained conditions (Fisher's test, p = 4.143×10^{-4}). Subject M showed a significant increase across the behavioural session for Novel trials (Spearman Correlation, r = 0.2805, p = 7.896×10^{-4}), but no change for Overtrained trials (Spearman Correlation, r = -0.0995, p = 0.2419). There was a significant difference between Novel and Overtrained conditions (Fisher's test, p = 0.0013). (B) Logistic fit of the probability of fixating the left stimulus first as a function of the value difference between the left and right stimuli for Novel trials. The data is split by the stage within the session. The inset shows the regression coefficient quantifying the effect of value on fixation direction at each stage of the session, with standard error. Subject F's first fixations became significantly move influenced by value as the session progressed (Spearman Correlation r = 0.9152 p = 4.667×10^{-4}). Subject M's first fixations also became significantly move influenced by value as the session progressed (r = 0.8667, p = 0.0027). C-D) As in (A-B), except for Subject M.

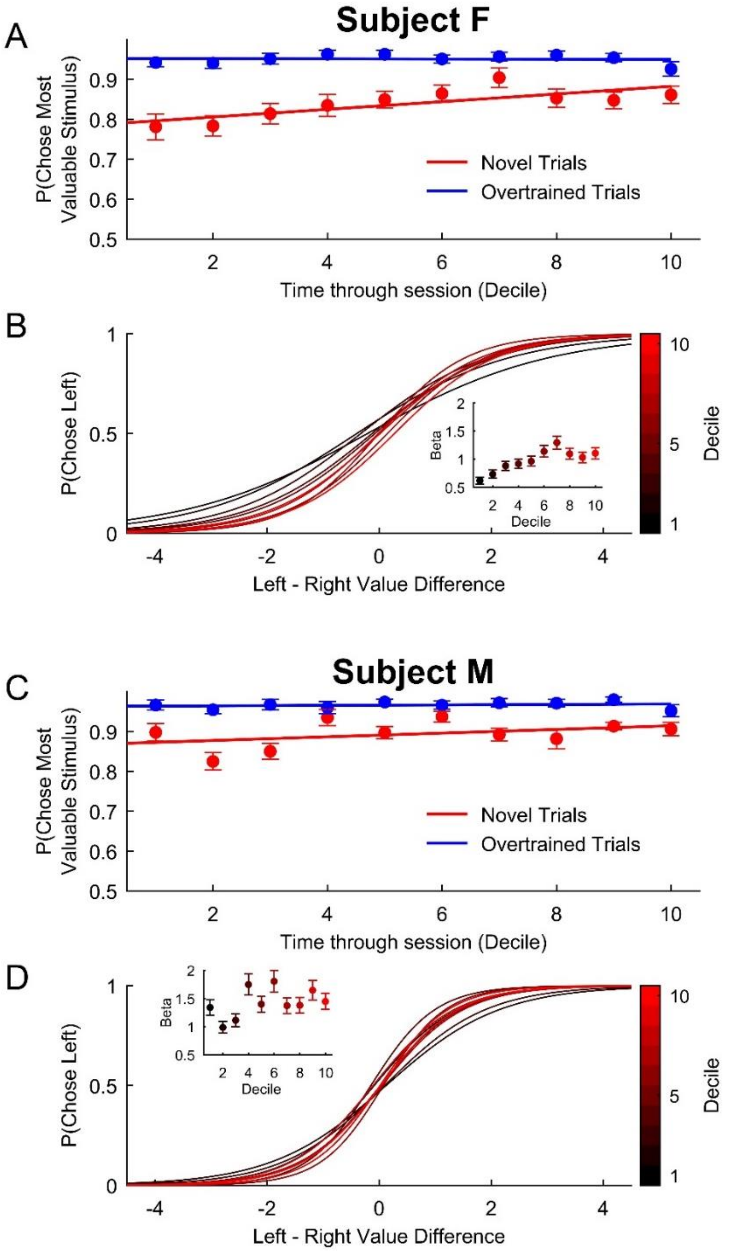

Fig. S6 Subject choice performance across a behavioural session

Choice performance on Novel trials is high throughout the session, even at the start of the choice phase. (A) The mean (±SEM) proportion of correct choices within each session for each trial decile is plotted for the Novel and Overtrained condition. Subject F showed a significant increase in performance across the behavioural session for Novel trials (Spearman Correlation, $r = 0.2427$, $p = 5.361 \times 10^{-4}$), but Overtrained accuracy did not change (Spearman Correlation, $r = 0.0262$, $p = 0.7125$). Subject M showed a weak non-significant increase in performance across the behavioural session for Novel trials (Spearman Correlation, $r = 0.1413$, $p = 0.0958$), but Overtrained accuracy did not change (Spearman Correlation, $r = 0.0435$, $p = 0.6101$). (B) Logistic fit of the probability of choosing the left stimulus as a function of the value difference between the left and right stimuli for Novel trials. Data is split by the stage within the session. The inset shows the regression coefficient quantifying the effect of value on choice at each stage of the session, with standard error. Subject F's choices became significantly move influenced by value as the session progressed (Spearman Correlation $r = 0.8061$ $p = 0.0082$). There was no correlation for Subject M ($r = 0.5394$, $p = 0.1133$). C-D) As in (A-B), except for Subject M.

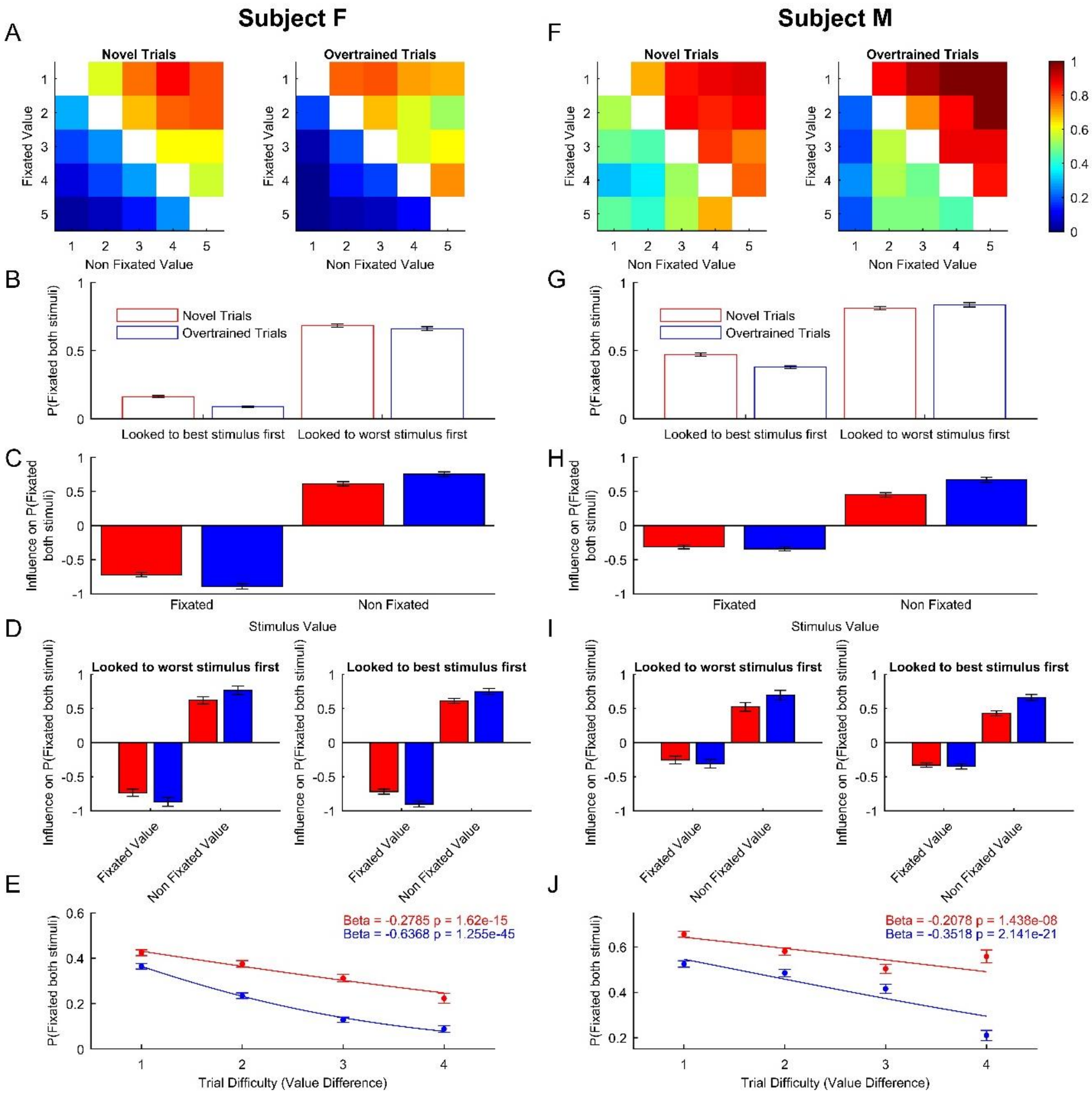

Fig. S7 Subjects' likelihood of fixating a second stimulus is influenced by covert evaluation of the unfixated stimulus

(A) The mean proportion of trials where Subject F viewed both stimuli, as a function of the initially fixated and unfixated stimuli values. Both the fixated and non-fixated stimulus values affect the likelihood of fixating the second stimulus. (B) The mean (±S.E) proportion of trials Subject F viewed both stimuli, as a function of whether the initially fixated stimulus was the more valuable. Subjects are more likely to view both stimuli when they look to the lower value stimulus first (F: $\chi^2(1)_{(Novel)}$ = 1077.8, $\chi(1)^2_{(Overtrained)}$ = 1352.1, M: $\chi(1)^2_{(Novel)}$ = 341.5, $\chi(1)^2_{(Overtrained)}$ = 380.6, $p<10^{-10}$ for all comparisons). (C) Logistic regression coefficients (±S.E) of the probability of fixating both stimuli as a function of the value of the fixated and non-fixated stimulus (Subject F: $T(7669)_{(Novel\ Fixated)}$= -23.55, $T(7669)_{(Novel\ Non\text{-}fixated)}$= 20.02, $T(7669)_{(Overtrained\ Fixated)}$= -24.00, $T(7669)_{(Overtrained\ Non\text{-}fixated)}$= 21.66; Subject M: $T(6278)_{(Novel\ Fixated)}$= -10.75, $T(6278)_{(Novel\ Non\text{-}fixated)}$= 14.32, $T(6278)_{(Overtrained\ Fixated)}$= -10.51, $T(6278)_{(Overtrained\ Non\text{-}fixated)}$= 18.44, $p<10^{-10}$ for all comparisons). Importantly, the value of the non-fixated stimulus significantly influences the probability of making a second fixation, suggesting it must have been covertly evaluated. (D) The regression analysis was repeated, split by whether Subject F initially viewed the more valuable stimulus. Regardless of whether the subject viewed the best stimulus first, both the fixated and the non-fixated value significantly influenced the probability of fixating the second stimulus. (E) The mean (±S.E) proportion of trials Subject F viewed both stimuli, as a function of trial difficulty. Subjects were more likely to fixate both stimuli on more difficult trials. Lines show a logistic regression fit, coefficients are shown in the figure panel. F-J) As in (A-E), except for Subject M.

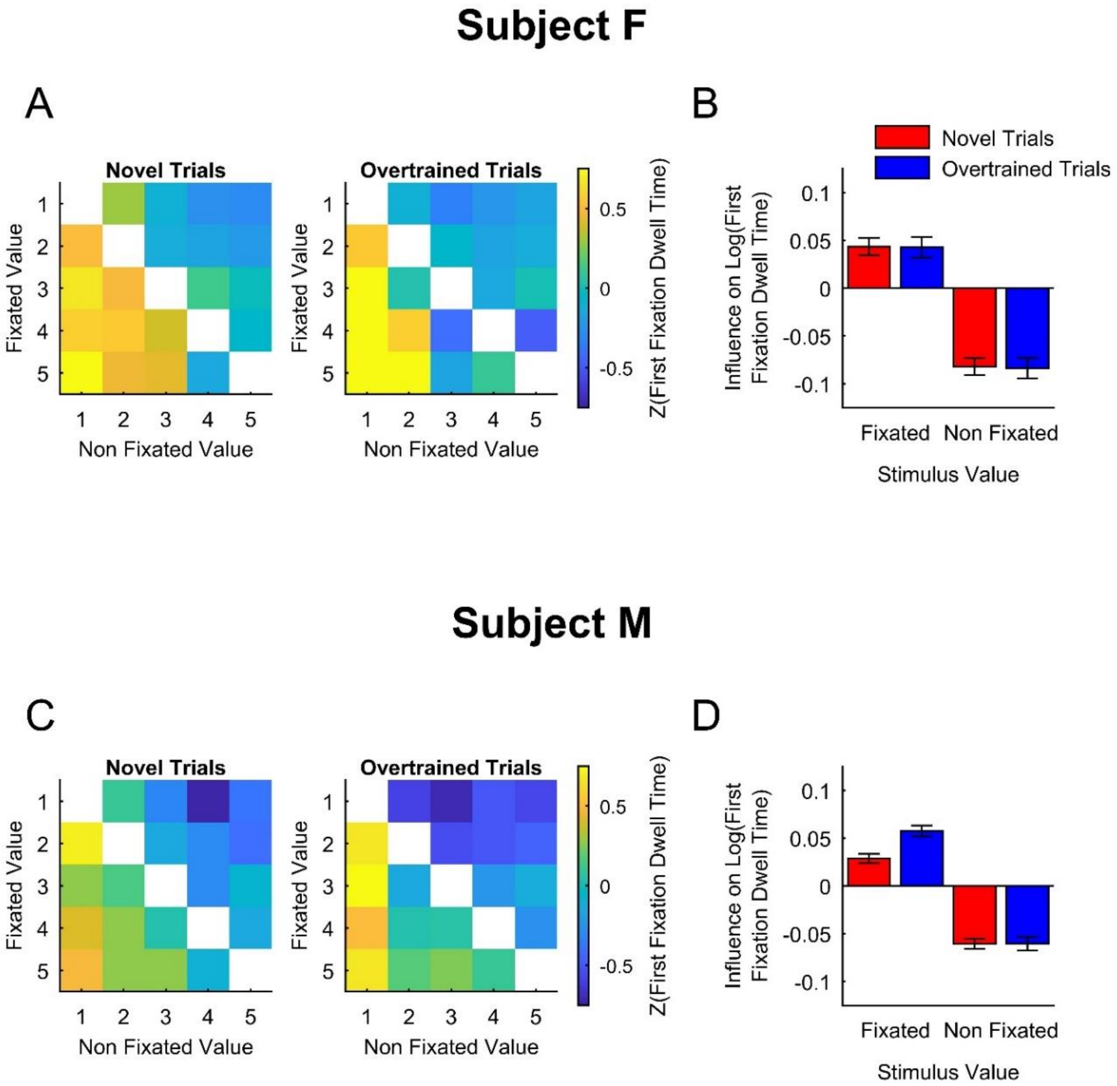

Fig. S8 Fixation Dynamics: Subjects' dwell time on the initially fixated stimulus is influenced by covert evaluation of the unfixated stimulus

(A) The z-scored dwell time on the first fixated stimulus on trials where Subject F view both stimuli. The dwell time is a function of the values of the initially fixated and unfixated stimuli. Subjects tend to dwell longer on higher value stimuli. However, the presence of a high value non-fixated alternative will reduce dwell time. (B) Linear regression coefficients (±S.E) of the log-transformed first stimulus dwell time as a function of the value of the fixated and non-fixated stimulus (Subject F: $T(2363)_{(\text{Novel Fixated})}= 4.88$, $T(2363)_{(\text{Novel Non-fixated})}= -9.17$, $T(2363)_{(\text{Overtrained Fixated})}= 3.90$, $T(2363)_{(\text{Overtrained Non-fixated})}= -7.74$; Subject M: $T(3296)_{(\text{Novel Fixated})}= 5.99$, $T(3296)_{(\text{Novel Non-fixated})}= -11.48$, $T(3296)_{(\text{Overtrained Fixated})}= 9.97$, $T(3296)_{(\text{Overtrained Non-fixated})}= -8.61$, $p<10^{-3}$ for all comparisons). Importantly, the value of the non-fixated stimulus significantly influences the dwell time on the fixated stimulus, suggesting it must have been covertly evaluated. C-D) As in (A-B), except for Subject M.

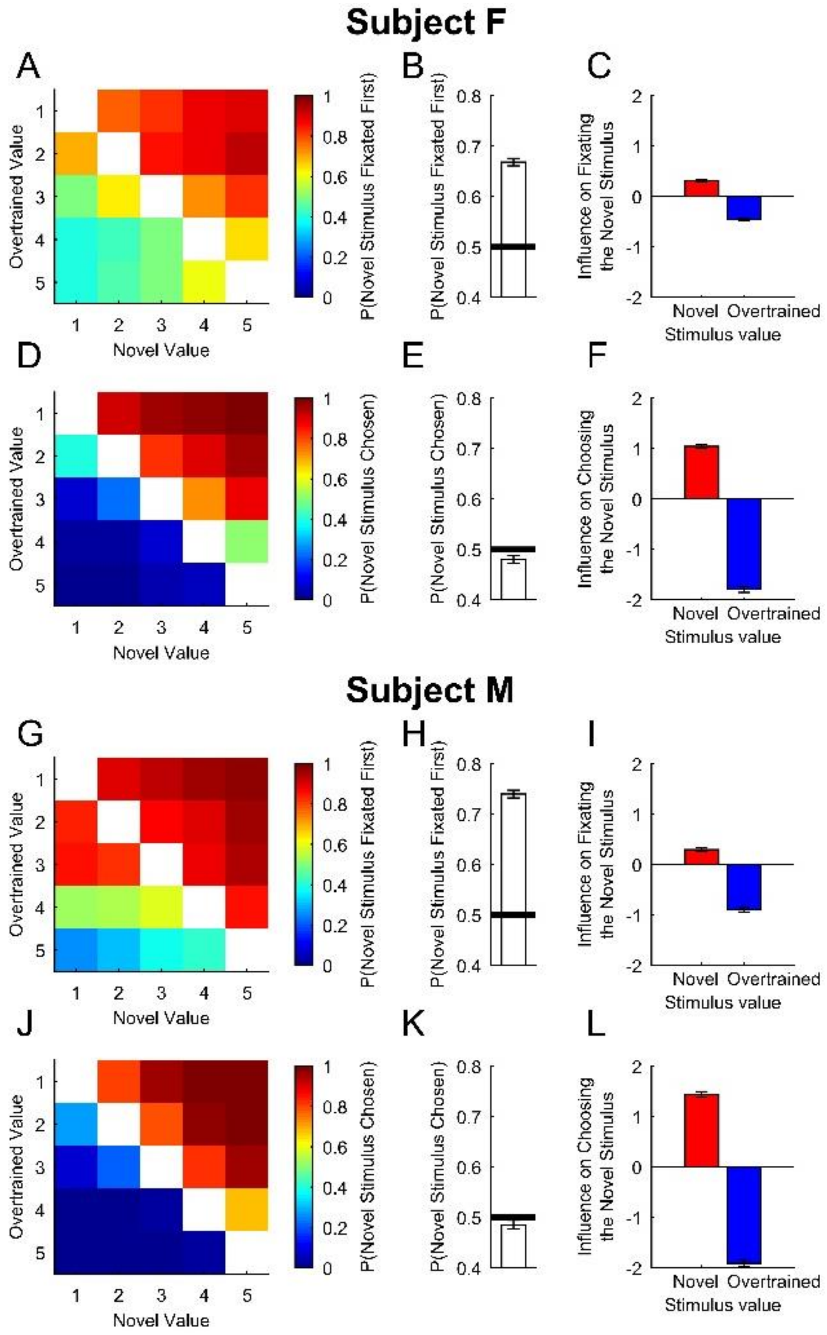

Fig. S9 Dissociation between fixation and choice behaviour on Mixed trials, for each subject

When presented with a choice between a Novel and an Overtrained stimulus, subjects have a preference to fixate the Novel stimulus first, but not to choose it. (A) The mean proportion of trials where the subject fixated the Novel stimulus first, as a function of the Novel and Overtrained stimuli values. Subjects had a preference to fixate the Novel stimulus first, but fixations were still influenced by the value of the stimuli. (B) Proportion of trials where the subject viewed the Novel stimulus first. Errorbars denote standard error. (C) Logistic regression coefficients for the influence of stimulus value on the probability of fixating the Novel stimulus first (Subject F: $T(3908)_{(Novel)}= 11.87$, $T(3908)_{(Overtrained)}= -16.40$; Subject M: $T(3210)_{(Novel)}= 8.50$, $T(3210)_{(Overtrained)}= -22.03$, $p<10^{-10}$ for all comparisons). Errorbars denote standard error. (D-F) As above, except for choice behaviour, rather than first fixation behaviour. There is no preference for choosing the Novel stimulus; choices are strongly influenced by value alone. Logistic regression coefficients for the influence of stimulus value on the probability of choosing the Novel stimulus (Subject F: $T(3919)_{(Novel)}= 25.50$, $T(3919)_{(Overtrained)}= -30.06$; Subject M: $T(3214)_{(Novel)}= 25.50$, $T(3214)_{(Overtrained)}= -26.52$, $p<10^{-10}$ for all comparisons). (G-L) As in (A-F), except for Subject M.

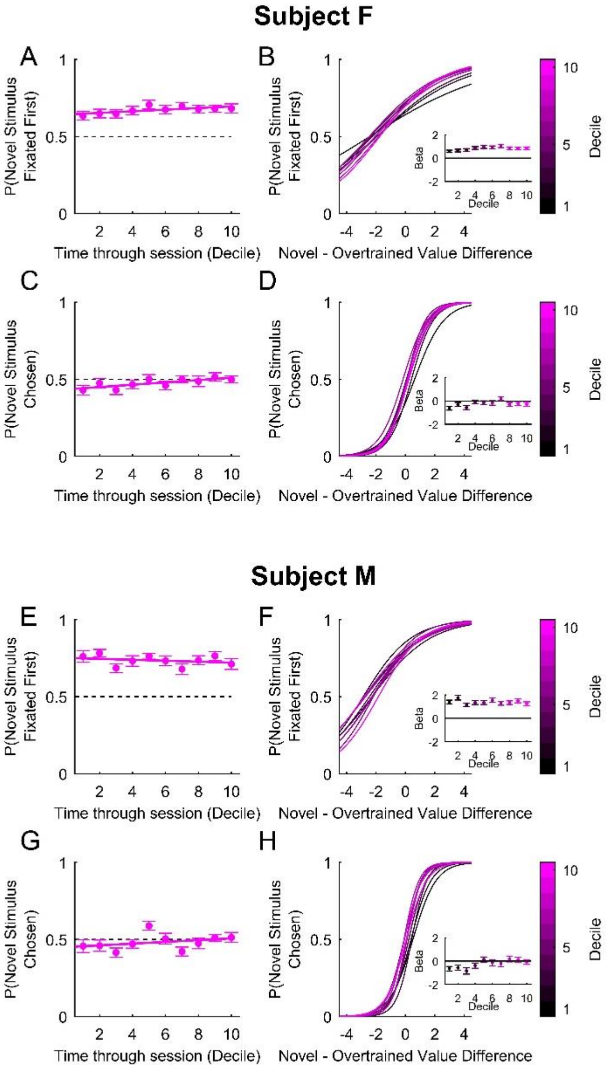

Fig. S10 The effect of stimulus novelty upon fixation and choice behaviour is relatively consistent across a behavioural session

(A, E) The mean (±SEM) proportion of Mixed trials where the subjects' first fixation was towards the Novel stimulus across sessions. (A) Subject F showed a weak trend towards becoming more likely to fixate the Novel stimulus first as the session progressed (Spearman Correlation, r = 0.1323, p = 0.0618). (E) Subject M's preference for fixating the Novel stimulus first did not change significantly across the behavioural session (Spearman Correlation, r = -0.0522, p = 0.5400). (B,F) The novelty bias was quantified using a complementary regression approach. Logistic fit of the probability of fixating the Novel stimulus first as a function of the value difference between the Novel and Overtrained stimuli, split by the stage within the session. The inset shows the constant term regression coefficients for each decile of trials (capturing the novelty bias), with standard error. A beta less than 0 indicates an Overtrained bias, a beta greater than 0 indicates a novelty bias. The fixation novelty bias quantified using regression was stable for both subjects (Spearman Correlation, Subject F r = 0.4667 p = 0.1782, Subject M r = -0.2970 p = 0.4070). (C,G) The mean (±SEM) proportion of trials where the subjects chose the Novel stimulus across sessions. Unlike first fixation behaviour, there is no bias to choose Novel stimuli. Both subjects showed a marginal preference for Overtrained stimuli at the start of the session. (C) Subject F became slightly more likely to choose the Novel stimulus first as the session progressed (Spearman Correlation, r = 0.1625, p = 0.0215). (G) Subject M's choices showed no significant change in novelty bias across the session (Spearman Correlation, r = 0.1377, p = 0.1047). (D,H) Logistic fit of the probability of choosing the Novel stimulus as a function of the value difference between the Novel and Overtrained stimuli, split by the stage within the session. The inset shows the constant term regression coefficients for each decile of trials (capturing the novelty bias), with standard error. When the change in novelty bias was calculated using the complementary regression method, Subject F's novelty preference did not change across the session (Spearman Correlation, r = 0.4182, p = 0.2324), and Subject M's increased slightly (Spearman Correlation, r = 0.7576, p = 0.0159).

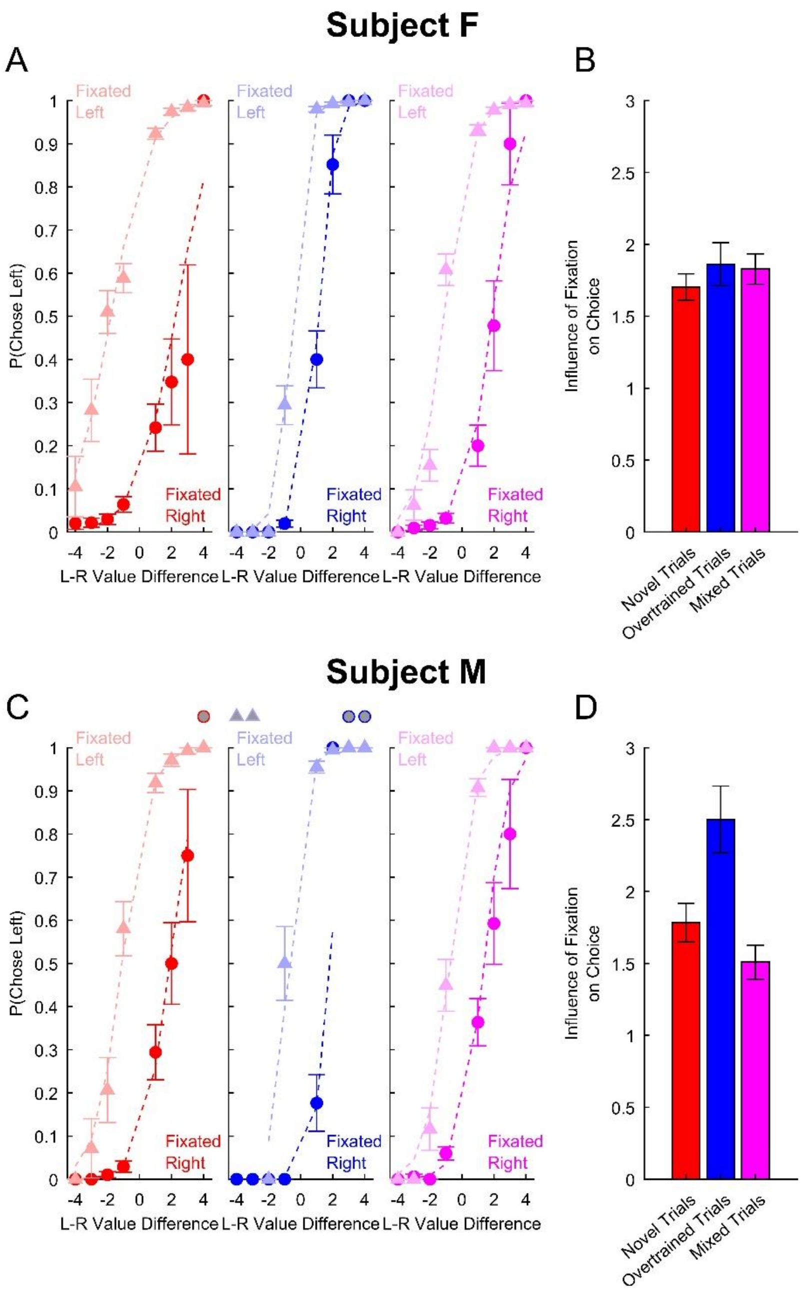

Fig. S11 Influence of fixation direction on economic choice for each subject on trials where a single stimulus is fixated

A) The influence of the fixation direction, over and above value difference. A separate plot shows Novel, Overtrained and Mixed trials. Markers show the proportion (±SE) of left choices at a specific value difference. The lightly shaded triangles show trials where the subject fixated left, the darker circular markers show when the subject fixated right. Dashed lines represent a logistic model fit (see **SI Appendix: Methods**). Subjects were more likely to choose the option they fixated. (B) Logistic regression coefficients (±SE) for a model predicting the economic choices of Subject F (Influence of Fixation: $T(7990)_{(Novel)}$= 18.49, $T(7990)_{(Overtrained)}$= 12.41, $T(7990)_{(Mixed)}$= 17.36, $p<10^{-10}$ for all comparisons). The direction fixated has a significant effect on choice over and above the stimulus values. (C-D) As in (A-B), except for Subject M. Logistic regression coefficients (±SE) for a model predicting economic choice (Influence of Fixation: $T(4574)_{(Novel)}$= 13.20, $T(4574)_{(Overtrained)}$= 10.77, $T(4574)_{(Mixed)}$= 12.82, $p<10^{-10}$ for all comparisons). Note, there are some conditions which do not occur for Subject M (e.g. Fixating right when the left value is much greater). These conditions without any observations are indicated by grey filled markers above the psychometric plots.

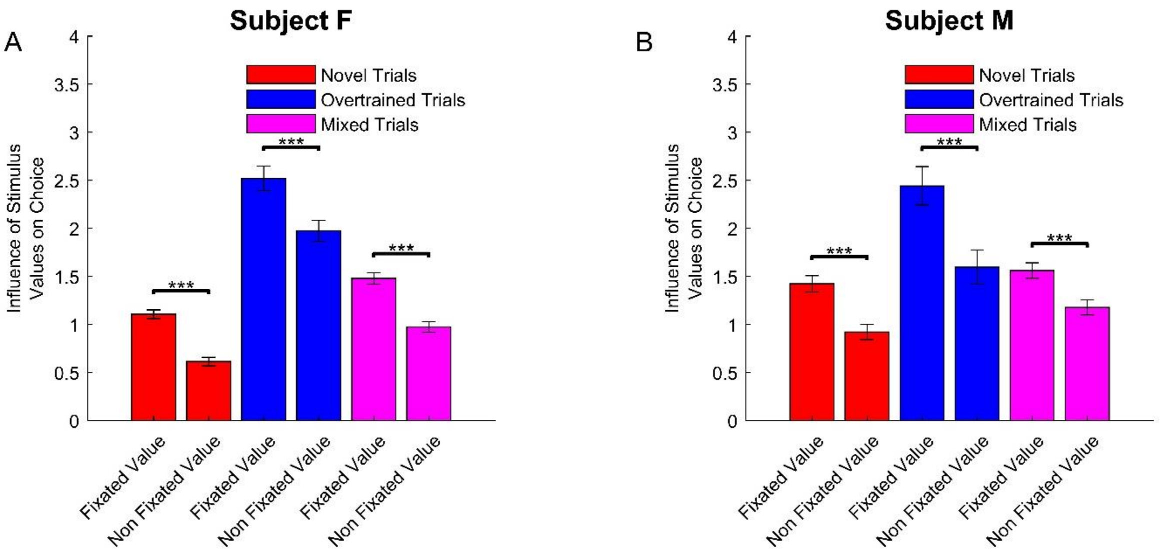

Fig. S12 Influence of fixated and non-fixated stimulus values on choices for each subject
A) Logistic regression coefficients (±S.E) of Subject F's economic choices as a function of the value of the fixated and non-fixated stimulus (see **SI Appendix: Methods**). Only trials where a single stimulus was fixated were analysed. The values of both stimuli significantly predicted choices (Subject F: $T(7990)_{(Novel\ Fixated)} = 24.02$, $T(7990)_{(Overtrained\ Fixated)} = 19.7$, $T(7990)_{(Mixed\ Fixated)} = 25.05$, $T(7990)_{(Novel\ Non\ Fixated)} = 13.59$, $T(7990)_{(Overtrained\ Non\ Fixated)} = 18.05$, $T(7990)_{(Mixed\ Non\ Fixated)} = 18.14$; Subject M: $T(4574)_{(Novel\ Fixated)} = 16.83$, $T(4574)_{(Overtrained\ Fixated)} = 12.16$, $T(4574)_{(Mixed\ Fixated)} = 19.37$, $T(4574)_{(Novel\ Non\ Fixated)} = 11.83$, $T(4574)_{(Overtrained\ Non\ Fixated)} = 9.008$, $T(4574)_{(Mixed\ Non\ Fixated)} = 15.1$; $p<10^{-10}$ for all comparisons). Importantly, the value of the non-fixated stimulus significantly influences choices, suggesting it must have been covertly evaluated. Furthermore, the fixated value had a stronger influence on choices (linear hypothesis test of $\beta_{(Fixated)} > \beta_{(Non\ Fixated)}$, $p<10^{-10}$ for all comparisons). B) As in (A), except for Subject M.

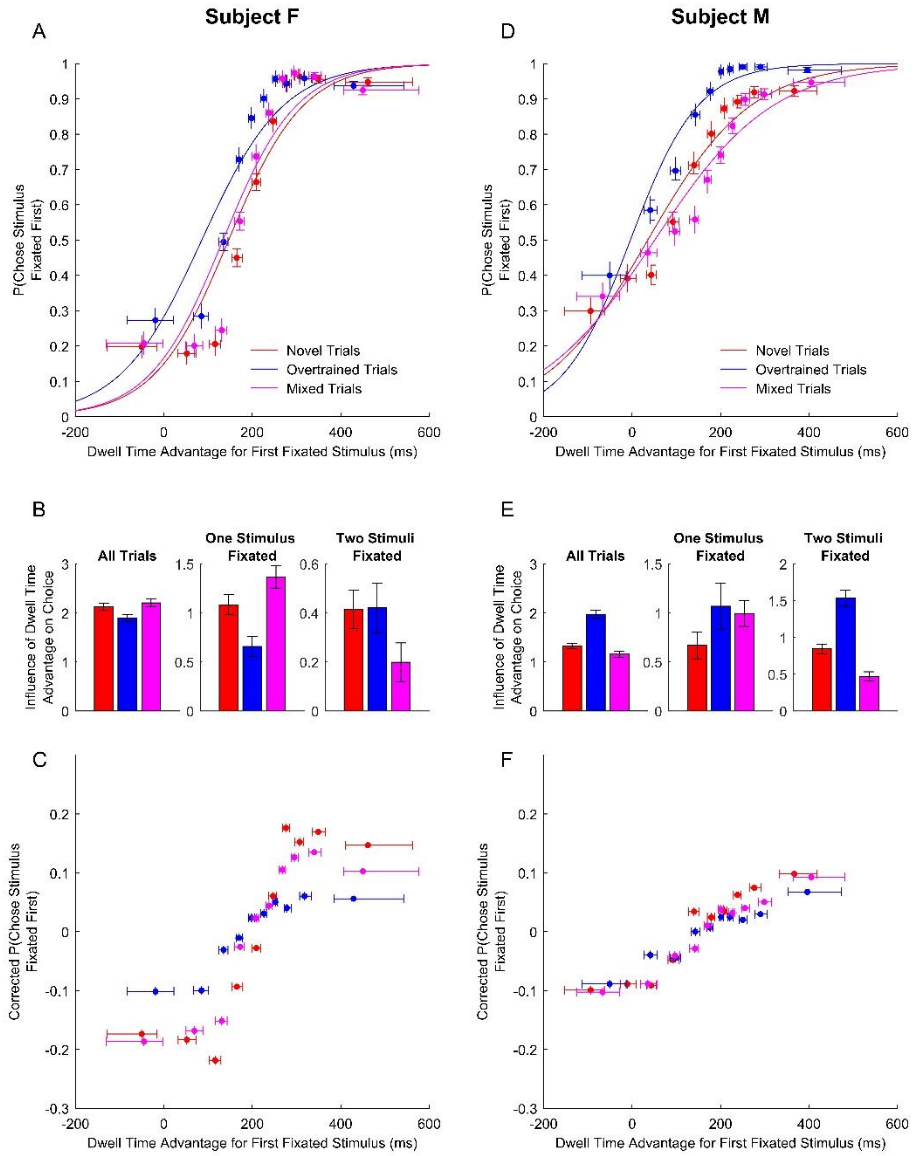

Fig. S13 Influence of fixation duration on economic choice for each subject

A) The probability of choosing the item fixated first, as a function of the final dwell time advantage allocated to that stimulus (see **SI Appendix: Methods**). The dwell times are binned into 10 deciles, with the mean choice probability for each bin indicated with a circular marker at the median dwell time of the bin. Lines show a logistic fit of the data. Horizontal errorbars show the interquartile range (i.e. central 50% values) for dwell times. Vertical errorbars show the standard error for choice probability. This analysis includes all trials for each condition type (i.e. Novel, Overtrained or Mixed). Subjects were more likely to choose the first fixated item when there was a greater time advantage in fixation duration allocated to it. (B) Logistic regression coefficients (±SE) for a model predicting economic choice as a function of z-scored dwell time advantage for the first stimulus. A separate panel is included for all trials, trials where a single stimulus was fixated, and trials where both stimuli were fixated. All Trials (Subject F: $T(11588)_{(Novel)}$= 28.90, $T(11588)_{(Overtrained)}$= 24.42, $T(11588)_{(Mixed)}$= 27.69; Subject M: $T(9492)_{(Novel)}$= 22.89, $T(9492)_{(Overtrained)}$= 22.39, $T(9492)_{(Mixed)}$= 20.31; $p<10^{-10}$ for all comparisons). One stimulus fixated trials (Subject F: $T(7993)_{(Novel)}$= 10.52, $T(7993)_{(Overtrained)}$= 6.53, $T(7993)_{(Mixed)}$= 11.81, $p<10^{-10}$ for all comparisons; Subject M: $T(4577)_{(Novel)}$= 4.924, $T(4577)_{(Overtrained)}$= 4.523, $T(4577)_{(Mixed)}$= 7.532, $p_{(Novel)}$= $8.464x10^{-7}$, $p_{(Overtrained)}$= $6.101x10^{-6}$, $p_{(Mixed)}<10^{-10}$). Both stimuli fixated trials (Subject F: $T(3580)_{(Novel)}$= 5.272, $T(3580)_{(Overtrained)}$= 4.175, $T(3580)_{(Mixed)}$= 2.524, $p_{(Novel)}$=$1.349x10^{-7}$, $p_{(Overtrained)}$=$2.975x10^{-5}$, $p_{(Mixed)}$= 0.01159; Subject M: $T(4908)_{(Novel)}$= 13.34, $T(4908)_{(Overtrained)}$= 13.98, $T(4908)_{(Mixed)}$= 7.669, $p<10^{-10}$ for all comparisons). (C) Analogous to A, except subtracting the probability of choosing the first fixated stimulus for each value difference (Fixated minus non-fixated value). This controls for any possible influence of the stimulus values on the fixation durations and shows that there remains a strong effect of total fixation time on choice. D-F) As in (A-C), except for Subject M.

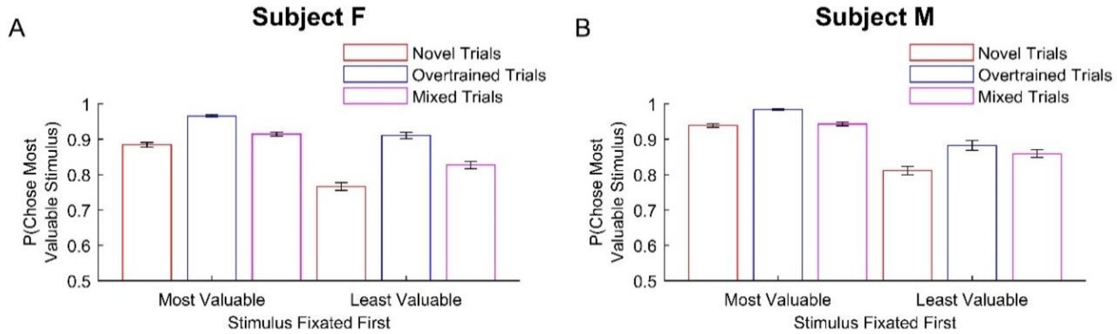

Fig. S14 Subjects' likelihood of choosing the correct stimulus is influenced by the first fixated stimulus
(A) The mean (±S.E) proportion of trials Subject F chose the most valuable stimulus, as a function of whether the initially fixated stimulus was the more valuable. Subjects are more likely to choose correctly when they look to the higher value stimulus first (Subject F: $\chi^2(1)_{(Novel)} = 94.43$, $\chi(1)^2_{(Overtrained)} = 49.75$, $\chi^2(1)_{(Mixed)} = 66.12$; Subject M: $\chi(1)^2_{(Novel)} = 121.5$, $\chi(1)^2_{(Overtrained)} = 146.4$, $\chi^2(1)_{(Mixed)} = 63.68$; $p<10^{-10}$ for all comparisons). B) As in (A), except for Subject M.

Trial Type	Proportion Correct (s.e)	
	Subject F	Subject M
Novel Probability Trials	0.817 (0.00877)	0.872 (0.00844)
Novel Magnitude Trials	0.861 (0.00792)	0.917 (0.007)
Overtrained Probability Trials	0.934 (0.00563)	0.962 (0.00482)
Overtrained Magnitude Trials	0.967 (0.00407)	0.972 (0.00414)
Mixed Probability Trials	0.872 (0.00756)	0.908 (0.00724)
Mixed Magnitude Trials	0.897 (0.00686)	0.924 (0.00658)
All Novel Trials	0.838 (0.00593)	0.894 (0.0055)
All Overtrained Trials	0.951 (0.00348)	0.967 (0.00317)
All Mixed Trials	0.884 (0.0051)	0.916 (0.00489)

Table S1: Proportion of correct choices for each trial condition. The proportion of choices where the most valuable stimulus was chosen, with standard error, is displayed for each subject. Values are rounded to 3 significant figures.

	Free Parameters	BIC		Mean Cross-Validated Log-likelihood	
		Subject F	Subject M	Subject F	Subject M
Model 1	9 (Constant, Left-Right Value Difference, Direction fixated; for each trial type)	2682.0	1234.6	-656.9	-296.6
Model 2	6 (Constant, Left-Right Value Difference; for each trial type)	3758.2	1823.2	-930.3	-447.8

Table S2: Model comparison results for logistic regressions predicting final choice. Two models were fit to predict subjects' choices on trials where a single stimulus was fixated (**Fig. 6A-B**). The Bayesian information criterion (BIC) and cross-validated log-likelihood (see **SI Appendix: Methods**) were calculated for each model. Model 1 is the best performing (i.e. higher likelihood, lower BIC) for both metrics, in both subjects. This means the direction fixated has an important impact on what subjects choose – even when controlling for stimulus values. Values are rounded to 1 decimal place.

**Figure 1D, Figure S2: Logistic Regression**	
**Response Variable**	
Left chosen	
**Regressor**	**Range**
Overtrained Trial	(0 or 1)
Novel Trial	(0 or 1)
Overtrained Left-Right Value Difference	(-4 to 4; or 0 if Novel trial)
Novel Left-Right Value Difference	(-4 to 4; or 0 if Overtrained trial)

**Figure 2C, Figure S3: Logistic Regression**	
**Response Variable**	
Left fixated first	
**Regressor**	**Range**
Overtrained Trial	(0 or 1)
Novel Trial	(0 or 1)
Overtrained Left-Right Value Difference	(-4 to 4; or 0 if Novel trial)
Novel Left-Right Value Difference	(-4 to 4; or 0 if Overtrained trial)

**Figure 3B, Figure S5: Logistic Regression**	
**Response Variable**	
Left fixated first	
**Regressor**	**Range**
Novel Trial was in first decile of recorded session	(0 or 1)
Novel Trial was in second decile of recorded session	(0 or 1)
…	(0 or 1)
Novel Trial was in tenth decile of recorded session	(0 or 1)
Novel Left-Right Value Difference (1st decile trials)	(-4 to 4; or 0 if trial not in 1st decile)
Novel Left-Right Value Difference (2nd decile trials)	(-4 to 4; or 0 if trial not in 2nd decile)
…	(-4 to 4) ; or 0 if trial not in n^{th} decile)
Novel Left-Right Value Difference (10th decile trials)	(-4 to 4; or 0 if trial not in 10th decile)

**Figure 4B, Figure S7: Logistic Regression**	
**Response Variable**	
Both stimuli fixated	
**Regressor**	**Range**
Overtrained Trial	(0 or 1)
Novel Trial	(0 or 1)
Overtrained Initially fixated value	(-2 to 2; or 0 if Novel trial)
Overtrained Initially unfixated value	(-2 to 2; or 0 if Novel trial)
Novel Initially fixated value	(-2 to 2; or 0 if Overtrained trial)
Novel Initially unfixated value	(-2 to 2; or 0 if Overtrained trial)

**Figure 4D, Figure S8: Linear Regression**	
**Response Variable**	
Log-transformed first stimulus dwell time (Both stimuli fixated trials only)	
**Regressor**	**Range**
Overtrained Trial	(0 or 1)
Novel Trial	(0 or 1)
Overtrained Initially fixated value	(-2 to 2; or 0 if Novel trial)
Overtrained Initially unfixated value	(-2 to 2; or 0 if Novel trial)
Novel Initially fixated value	(-2 to 2; or 0 if Overtrained trial)
Novel Initially unfixated value	(-2 to 2; or 0 if Overtrained trial)

**Figure 5C, Figure S9: Logistic Regression**	
**Response Variable**	
Novel stimulus fixated first	
**Regressor**	**Range**
Constant term	(1)
Overtrained Value	(1 to 5)
Novel Value	(1 to 5)

**Figure 5F, Figure S9: Logistic Regression**	
**Response Variable**	
Novel stimulus chosen	
**Regressor**	**Range**
Constant term	(1)
Overtrained Value	(1 to 5)
Novel Value	(1 to 5)

Figure 6A-B, Figure S11, Table S2 Full Model: Logistic Regression	
**Response Variable**	
Left chosen (One stimuli fixated trials only)	
**Regressor**	**Range**
Overtrained Trial	(0 or 1)
Novel Trial	(0 or 1)
Mixed Trial	(0 or 1)
Overtrained Left-Right Value Difference	(-4 to 4; or 0 if not Overtrained trial)
Novel Left-Right Value Difference	(-4 to 4; or 0 if not Novel trial)
Mixed Left-Right Value Difference	(-4 to 4; or 0 if not Mixed trial)
Overtrained Direction Fixated	(-1 fixated right; +1 fixated left; 0 if not Overtrained trial)
Novel Direction Fixated	(-1 fixated right; +1 fixated left; 0 if not Novel trial)
Mixed Direction Fixated	(-1 fixated right; +1 fixated left; 0 if not Mixed trial)

Figure 6C, Figure S12: Logistic Regression	
**Response Variable**	
Left chosen (One stimuli fixated trials only)	
**Regressor**	**Range**
Overtrained Trial	(0 or 1)
Novel Trial	(0 or 1)
Mixed Trial	(0 or 1)
Overtrained Fixated Value Regressor	(-5 to 5; +Fixated value if looked left, -Fixated value if looked right, or 0 if not Overtrained trial)
Novel Fixated Value Regressor	(-5 to 5; +Fixated value if looked left, -Fixated value if looked right, or 0 if not Novel trial)
Mixed Fixated Value Regressor	(-5 to 5; +Fixated value if looked left, -Fixated value if looked right, or 0 if not Mixed trial)
Overtrained Non-Fixated Value Regressor	(-5 to 5; -Non-Fixated value if looked left, +Non-Fixated value if looked right, or 0 if not Overtrained trial)
Novel Non-Fixated Value Regressor	(-5 to 5; -Non-Fixated value if looked left, +Non-Fixated value if looked right, or 0 if not Novel trial)
Mixed Non-Fixated Value Regressor	(-5 to 5; -Non-Fixated value if looked left, +Non-Fixated value if looked right, or 0 if not Mixed trial)

Figure 6D, Figure S13: Logistic Regression	
**Response Variable**	
Stimulus fixated first chosen	
**Regressor**	**Range**
Overtrained Trial	(0 or 1)
Novel Trial	(0 or 1)
Mixed Trial	(0 or 1)
Overtrained Dwell Time Regressor	Z-scored (Total dwell time on first fixated stimulus – total dwell time on other stimulus); 0 if not Overtrained trial
Novel Dwell Time Regressor	Z-scored (Total dwell time on first fixated stimulus – total dwell time on other stimulus); 0 if not Novel trial
Mixed Dwell Time Regressor	Z-scored (Total dwell time on first fixated stimulus – total dwell time on other stimulus); 0 if not Mixed trial

Figure S2C, G: Linear Regression	
**Response Variable**	
Log-transformed reaction time (Overtrained Trials only)	
**Regressor**	**Range**
Constant Term	(1)
Overtrained value difference (i.e. Trial Difficulty)	(1 to 4)
**Response Variable**	
Log-transformed reaction time (Novel Trials only)	
**Regressor**	**Range**
Constant Term	(1)
Novel value difference (i.e. Trial Difficulty)	(1 to 4)

**Figure S3E, J: Linear Regression**	
**Response Variable**	
Log-transformed latency of first fixation (Overtrained Trials only)	
**Regressor**	**Range**
Constant Term	(1)
Overtrained Value Difference (i.e. Trial Difficulty)	(1 to 4)
**Response Variable**	
Log-transformed latency of first fixation (Novel Trials only)	
**Regressor**	**Range**
Constant Term	(1)
Novel Value Difference (i.e. Trial Difficulty)	(1 to 4)

**Fig. S4B, D: Logistic Regression**	
**Response Variable**	
Most valuable stimulus fixated first	
**Regressor**	**Range**
Overtrained Trial	(0 or 1)
Novel Trial	(0 or 1)
Mixed Trial	(0 or 1)
Overtrained Lower Value Stimulus	(-1.5 to 1.5; or 0 if not Overtrained trial)
Novel Lower Value Stimulus	(-1.5 to 1.5; or 0 if not Novel trial)
Mixed Lower Value Stimulus	(-1.5 to 1.5; or 0 if not Mixed trial)

**Figure S6C, D: Logistic Regression**	
**Response Variable**	
Left chosen	
**Regressor**	**Range**
Novel Trial was in first decile of recorded session	(0 or 1)
Novel Trial was in second decile of recorded session	(0 or 1)
…	(0 or 1)
Novel Trial was in tenth decile of recorded session	(0 or 1)
Novel Left-Right Value Difference (1st decile trials)	(-4 to 4; or 0 if trial not in 1st decile)
Novel Left-Right Value Difference (2nd decile trials)	(-4 to 4; or 0 if trial not in 2nd decile)
…	(-4 to 4) ; or 0 if trial not in n^{th} decile)
Novel Left-Right Value Difference (10th decile trials)	(-4 to 4; or 0 if trial not in 10th decile)

**Fig. S7E, J: Logistic Regression**	
**Response Variable**	
Both stimuli fixated	
**Regressor**	**Range**
Overtrained Trial	(0 or 1)
Novel Trial	(0 or 1)
Overtrained Value Difference (i.e. Trial Difficulty)	(-1.5 to 1.5; or 0 if Novel trial)
Novel Value Difference (i.e. Trial Difficulty)	(-1.5 to 1.5; or 0 if Overtrained trial)

**Figure S10B, F: Logistic Regression**	
**Response Variable**	
Novel fixated first	
**Regressor**	**Range**
Mixed Trial was in first decile of recorded session	(0 or 1)
Mixed Trial was in second decile of recorded session	(0 or 1)
…	(0 or 1)
Mixed Trial was in tenth decile of recorded session	(0 or 1)
Mixed Novel-Overtrained Value Difference (1st decile trials)	(-4 to 4; or 0 if trial not in 1st decile)
Mixed Novel-Overtrained Value Difference (2nd decile trials)	(-4 to 4; or 0 if trial not in 2nd decile)
…	(-4 to 4) ; or 0 if trial not in n^{th} decile)
Mixed Novel-Overtrained Value Difference (10th decile trials)	(-4 to 4; or 0 if trial not in 10th decile)

**Figure S10D, H: Logistic Regression**	
**Response Variable**	
Novel chosen	
**Regressor**	**Range**
Mixed Trial was in first decile of recorded session	(0 or 1)
Mixed Trial was in second decile of recorded session	(0 or 1)
…	(0 or 1)
Mixed Trial was in tenth decile of recorded session	(0 or 1)
Mixed Novel-Overtrained Value Difference (1st decile trials)	(-4 to 4; or 0 if trial not in 1st decile)
Mixed Novel-Overtrained Value Difference (2nd decile trials)	(-4 to 4; or 0 if trial not in 2nd decile)
…	(-4 to 4) ; or 0 if trial not in n^{th} decile)
Mixed Novel-Overtrained Value Difference (10th decile trials)	(-4 to 4; or 0 if trial not in 10th decile)

Table S3: Details of regression models. The full regression model testing the impact of the direction fixated, over and above stimulus value (**Fig. 6A-B**), is shown in this table. A further model containing a subset of its predictors was compared using cross-validation. The predictors used in this model are detailed in **Table S2**.

References

1. Hunt LT, *et al.* (2018) Triple dissociation of attention and decision computations across prefrontal cortex. *Nat Neurosci* 21(10):1471-1481.
2. Asaad WF & Eskandar EN (2008) A flexible software tool for temporally-precise behavioral control in Matlab. *J Neurosci Methods* 174(2):245-258.
3. Asaad WF & Eskandar EN (2008) Achieving behavioral control with millisecond resolution in a high-level programming environment. *J Neurosci Methods* 173(2):235-240.
4. Asaad WF, Santhanam N, McClellan S, & Freedman DJ (2013) High-performance execution of psychophysical tasks with complex visual stimuli in MATLAB. *J Neurophysiol* 109(1):249-260.
5. Engbert R & Mergenthaler K (2006) Microsaccades are triggered by low retinal image slip. *Proc. Natl. Acad. Sci. U. S. A.* 103(18):7192-7197.
6. Engbert R & Kliegl R (2003) Microsaccades uncover the orientation of covert attention. *Vision Res* 43(9):1035-1045.
